# Diversity and distribution of genetic variation in gammarids: Comparing patterns between invasive and non‐invasive species

**DOI:** 10.1002/ece3.3208

**Published:** 2017-08-22

**Authors:** Miguel Baltazar‐Soares, Filipa Paiva, Yiyong Chen, Aibin Zhan, Elizabeta Briski

**Affiliations:** ^1^ GEOMAR, Helmholtz‐Zentrum für Ozeanforschung Kiel Kiel Germany; ^2^ Faculty of Science and Technology Bournemouth University Poole Dorset United Kingdom of Great Britain and Northern Ireland; ^3^ Research Center for Eco‐Environmental Sciences Chinese Academy of Sciences Beijing China

**Keywords:** aquatic invasive species, biological invasions, Gammaridae, genetic diversity, population differentiation

## Abstract

Biological invasions are worldwide phenomena that have reached alarming levels among aquatic species. There are key challenges to understand the factors behind invasion propensity of non‐native populations in invasion biology. Interestingly, interpretations cannot be expanded to higher taxonomic levels due to the fact that in the same genus, there are species that are notorious invaders and those that never spread outside their native range. Such variation in invasion propensity offers the possibility to explore, at fine‐scale taxonomic level, the existence of specific characteristics that might predict the variability in invasion success. In this work, we explored this possibility from a molecular perspective. The objective was to provide a better understanding of the genetic diversity distribution in the native range of species that exhibit contrasting invasive propensities. For this purpose, we used a total of 784 sequences of the cytochrome *c* oxidase subunit I of mitochondrial DNA (mtDNA‐COI) collected from seven Gammaroidea, a superfamily of Amphipoda that includes species that are both successful invaders (*Gammarus tigrinus*,* Pontogammarus maeoticus,* and *Obesogammarus crassus*) and strictly restricted to their native regions (*Gammarus locusta*,* Gammarus salinus*,* Gammarus zaddachi,* and *Gammarus oceanicus*). Despite that genetic diversity did not differ between invasive and non‐invasive species, we observed that populations of non‐invasive species showed a higher degree of genetic differentiation. Furthermore, we found that both geographic and evolutionary distances might explain genetic differentiation in both non‐native and native ranges. This suggests that the lack of population genetic structure may facilitate the distribution of mutations that despite arising in the native range may be beneficial in invasive ranges. The fact that evolutionary distances explained genetic differentiation more often than geographic distances points toward that deep lineage divergence holds an important role in the distribution of neutral genetic diversity.

## 1 INTRODUCTION

Contemporary scenarios of species colonizing new habitats are explained by anthropogenically driven introductions and/or the ongoing shifts in climatic conditions (Capinha, Essl, Seebens, Moser, & Pereira, 2015; Hellmann, Byers, Bierwagen, & Dukes, 2008). While the former literally transport organisms from its natural distribution into non‐native ranges (Lockwood, Hoopes, & Marchetti, 2013), the latter promotes the expansion of natural boundaries following an extension of habitat optima. In the case of aquatic species, the increasing connectivity levels of human trade networks have placed shipping as the dominant vector of introductions (Keller, Drake, Drew, & Lodge, 2011). Introduction processes associated with shipping may occur due to the presence of living organisms in ballast waters and/or through the attachment of organisms to the hulls as part of the fouling community (Briski, Chan, MacIsaac, & Bailey, 2014; Sylvester et al., 2011). The result of such huge inter‐regional mixing of species is a patchy geographic distribution (Briski et al., 2013; Lockwood et al., 2013; Sylvester et al., 2011).

Through analyses of molecular data, invasion genetics aims at identifying the routes of biological invasions and the dispersal of non‐native species, as well as mechanisms underlying their success (Bock et al., 2015; Muirhead et al., 2008; Sherman et al., 2016). In this sense, genetic research is routinely used to characterize indices of diversity, identify source populations, discriminate between translocation events and/or invasive lineages, obtain indirect demographic estimates, or estimate neutral levels of population differentiation (Bock et al., 2015; Cristescu, 2015). All of these signatures are optimally inferred from genetic markers whose evolution is known to be neutral or near neutral, which may avoid direct confounding effects of natural selection (e.g., the effect of background selection in demographic inferences (Ewing & Jensen, 2016). Estimating the level of neutral population differentiation is a key process in invasion genetics as it allows building expectations on how adaptive variation evolves and contributes to invasive success (Colautti & Lau, 2015). The interpretation of fixation indices together with other metrics can be an important indicator to understand biological invasions. For example, in a recent study, Gaither, Bowen, and Toonen (2013) investigated whether *F*_ST_—a commonly used fixation index—and dispersal capacity could forecast invasion success (Gaither et al., 2013). The authors found that *F*_ST_ among populations in the native range negatively correlated with the geographic extent of spread (Gaither et al., 2013). Among continuously distributed populations (such as those within native range), neutral estimates of differentiation can often be explained by geographic distance among populations (Wright, 1943). This is because those estimates are proxies for migration rates across evolutionary time scales; by excluding selection and in the absence of migration, drift alone is responsible for the fixation of population‐specific variants (Nielsen & Slatkin, 2013). However, it has been shown that differentiation levels among introduced populations deviate from expectations built on linear relationships with geographic distance (Leblois, Rousset, Tikel, Moritz, & Estoup, 2000; Marrs, Sforza, & Hufbauer, 2008; Zhan et al., 2012). Aside from natural selection, several factors might provide the explanation for this discontinuities in the colonization process, such has, multiple colonization events, genetically distinct sources of introduction, and processes associated with founder effects (Bock et al., 2015; Estoup & Guillemaud, 2010; Excoffier & Ray, 2008; Roman & Darling, 2007).

During the Cretaceous periods, Gammaroidea—a large superfamily of Amphipoda (Hou & Sket, 2016)—underwent a massive diversification event in the Tethys region, resulting in the evolution of highly distinct lineages (Cristescu, Hebert, & Onciu, 2003; Hou, Sket, & Li, 2014). Phylogeographic analyses showed that further diversification occurred heterogeneously within each lineage and was accompanied by various levels of range expansion. For example, while *Gammarus* rapidly radiated across Eurasia and North America (Hou, Sket, Fišer, & Li, 2011), the lineage *Pontogammarus* remained restricted to the Tethyan Basin (Hou et al., 2014). Nowadays, these organisms are represented in nearly every type of aquatic environments and it is common to encounter the same species in highly distinct salinity ranges. It is therefore not surprising to find members of this superfamily among the records of successful invasive species (Casties, Seebens, & Briski, 2016; DAISIE, [Link]; GISD, 2017). One of the most prominent examples is *Gammarus tigrinus*, an amphipod native to saltwater habitats of North America that has invaded both fresh and brackish waters, such as the Laurentian Great Lakes and Baltic Sea (Ricciardi & MacIsaac, 2000). This example relates to human‐mediated introductions, and ship ballast water has been assumed as the most probable transport vector of such long‐range transoceanic expansions (Ricciardi & MacIsaac, 2000). At a much smaller geographic scale, but most likely also facilitated by human intervention, the native Ponto‐Caspian species *Pontogammarus maeoticus* and *Obesogammarus crassus* are expected to spread toward central and eastern Europe as examples of gradual invasions through rivers and canals (Bij de Vaate, Jazdzewski, Ketelaars, Gollasch, & Van der Velde, 2002; Cristescu et al., 2003; Pligin, Matchinskaya, Zheleznyak, & Linchuk, 2014; Semenchenko & Vezhnovetz, 2008). In contrast, some other gammarids such as *Gammarus locusta*,* Gammarus salinus*,* Gammarus zaddachi,* and *Gammarus oceanicus* are restricted to native regions and are all commonly found in the Baltic Sea (Herkül, Lauringson, & Kotta, 2016). Available literature involving genetic analyses of the *Gammarus* genus focuses mainly on two topics. The first one tests hypothesis of evolution, colonization, or expansion out of the Tethys Basin into North Europe, Asia, and North America due to a succession of geological events and more recently due to anthropogenic activities (Kelly, MacIsaac, & Heath, 2006; Kelly, Muirhead, Heath, & Macisaac, 2006; Ricciardi & MacIsaac, 2000). The second one focuses on taxonomical resolution through DNA barcoding, as it is hypothesized that numerous cryptic species exist within *Gammarus* genus (Costa, Henzler, Lunt, Whiteley, & Rock, 2009; Raupach et al., 2015).

In this study, we use a holistic approach to investigate the distribution of genetic diversity, through estimates of genetic indices and population differentiation, in the context of biological invasions. More specifically, we extend comparisons to species of the same genus. We focused on seven gammarid species chosen based on their variable invasive propensities: *G. tigrinus*,* P. maeoticus,* and *O. crassus* have established populations outside their native ranges, while *G. locusta*,* G. salinus, G. zaddachi*, and *G. oceanicus* are apparently restricted to their native ranges. For the sake of consistency, we will henceforth call *G. tigrinus*,* P. maeoticus,* and *O. crassus* as invasive and *G. locusta*,* G. salinus G. zaddachi*, and *G. oceanicus* as non‐invasive. We collected 12 populations distributed among species, sequenced the cytochrome *c* oxidase subunit I region of the mitochondria DNA (mtDNA‐COI), and complemented our sequences with available mtDNA‐COI sequences for each respective species from NCBI.

Despite that Gaither et al. (2013) reported that less structured populations are more likely to disperse/invade new habitats, we were not able to measure natural dispersal for any of the species in this study. Therefore, we hypothesize that population structure—as estimated by *F*_ST_—will be higher among native populations of noninvasive species. Furthermore, due to the recurrent identification of deep evolutionary lineages within this genus (Cristescu et al., 2003; Hou et al., 2014), we hypothesize that (1) owning to the result of long‐term natural microevolutionary processes, population differentiation will correlate preferentially with geographic distance in populations in their native ranges; and (2) as a result of contemporary human‐mediated introductions, population differentiation will correlate with evolutionary distances among populations in the introduced range.

## 2 METHODS

### 2.1 Sample field collection, amplification, sequencing, and data collection from the NCBI

Specimens of five species were collected in their native areas, whereas those of *G. tigrinus*, due to practicality and distance from available testing station, were collected in their invaded regions (Table S1). We amplified and sequenced the mtDNA‐COI of six gammarid species collected from Northern Europe and Ponto‐Caspian region: *G. tigrinus*,* P. maeoticus*,* G. locusta*,* G. salinus G. zaddachi*, and *O. crassus*. Genomic DNA was extracted from the telson of the organisms with the Marine Animal DNA Kit (TIANGEN; Beijing, China) following manufacturer's instruction. A fragment of the mtDNA‐COI was amplified using a few different pairs of primers: LCO1490 and HCO2198 (Vrijenhoek, 1994) and UCOIF and UCOIR (Costa et al., 2009), and for *G. tigrinus* species‐specific primers from Kelly, MacIsaac, et al. (2006), Kelly, Muirhead, et al. (2006). PCR amplifications were carried out in 20μl volume including 10 X Taq Buffer (containing MgCl_2_), 100 mmol/L dNTPs, 10 mmol/L of each primer, 1– 10 ng of genomic DNA, and 1 U of Taq DNA polymerase (Takara China; Dalian, China). The amplification protocol consisted of 5‐min denaturation at 94°C, followed by 33 cycles of denaturation at 94°C for 35 s, annealing at 47°C for 45 s, extension at 69° for 45 s, and a final extension step of 69°C for 10 min. PCR products were prepared for sequencing using a BigDye Terminator v3.1 cycle sequencing kit (Thermo Fisher Scientific, Waltham, USA), purified with a BigDye XTerminator Purification Kit (Thermo Fisher Scientific, Waltham, USA), and sequenced on an automated ABI 3130XL capillary sequencer. In order to complement our field samples,we retrieved available mtDNA‐COI sequences for our six species and one additional (i.e., *Gammarus oceanicus*) from NCBI.

### 2.2 Alignment and trimming and quality check of the sequences per species

The sequences of all species were treated in parallel. Downstream analyses were performed independently for each species. Alignments were performed in Muscle v3.8.31 with default conditions (Edgar, 2004). Sequences were trimmed to the same size within species after visual inspection in BioEdit v7.0.4.1 (Hall, 1999).

### 2.3 Genetic diversity indices and phylogenies

The number of haplotypes (*n*Hap), number of segregation sites (*S*), haplotype diversity (Hd), and nucleotide diversity (π) were calculated for each sampling location in DnaSP v5 (Librado & Rozas, 2009). first we compared the averages of all genetic diversity indices between native populations of invasive species (*G. tigrinus*,* P. maeoticus* and *O. crassus*) versus those of non‐invasive species (*G. salinus*,* G. oceanicus*,* G. zaddachi*,* G. locusta*). Nucleotide substitution model was estimated independently for each species through maximum‐likelihood method by allowing a strong branch swapping. Best‐fit model was chosen according to Bayesian inference criteria for downstream analyses. Phylogenetic relationships were investigated with the Neighbor‐joining method (Saitou & Nei, 1987) with the species‐specific substitution model as well as including transitions and transversions. Statistical support was inferred with 1,000 bootstraps. Neighbor‐joining trees were condensed to 75% bootstrap value with the objective of identifying deep divergent phylogenies. All analyses associated with phylogenetic inference and the construction of Neighbor‐joining trees were performed in MEGA v6.0 (Tamura, Stecher, Peterson, Filipski, & Kumar, 2013).

### 2.4 Population differentiation and evolutionary divergence

Population differentiation was estimated by calculating pairwise *F*_ST_ (Wright, 1943) based on haplotype frequencies between sampling localities in the software Arlequin v.3.5 using 10,000 permutations (Excoffier & Lischer, 2009). Statistical significance was assessed after corrections for multiple testing following the suggestions of Narum (2006). As evolutionary distances (*d*) are statistical estimates that aim at calculating the divergence between DNA lineages (Tamura & Kumar, 2002), we employed this method implemented in MEGA v6.0 (Tamura & Kumar, 2002) to calculate average evolutionary distances between pairwise sampling locations. We then considered this measure a proxy for variable representation of lineages between localities. This measure is distinctive from population differentiation such as the *F*_ST_, as the latter is directly related to the variance in allelic frequencies among populations and cannot be considered a distance measure (Holsinger & Weir, 2009; Wright, 1943).

### 2.5 Geographic distances and statistical models

Geographic distances (in kilometers) were calculated by comparing the latitude and longitude coordinates of each location through java scripts implemented in http://www.movable-type.co.uk/scripts/latlong.html. All geographic distances were log‐transformed to base 10 in order to normalize its distribution and approximate the values to the order of magnitude of those of *F*_ST_ and evolutionary distance. To test a possible relationship between population structuring and invasion propensity, we tested whether invasive (i.e., *G. tigrinus*,* P. maeoticus,* and *O. *crassus) and non‐invasive species (i.e., G. *locusta*,* G. salinus*,* G. oceanicus*,* G. zaddachi*) differed in the degree of population structuring at their native ranges. To account for a possible influence of spatial distance between sampling sites on the *F*_ST_ estimates, we first averaged the log geographic distances obtained per matrix and then divided all *F*_ST_ obtained through pairwise comparisons by that value. This procedure was performed independently for each species.

Finally, in order to explore whether geographic distance or evolutionary divergence better explains population structure, we built linear models with *F*_ST_ as the response variable and evolutionary divergence plus geographic distance as predictors. Under neutral evolution, measures of genetic differentiation, such as the *F*_ST_ estimates among continuous populations, are expected to increase linearly with geographic distances (i.e., isolation by distance (IBD); (Wright, 1943). Because of that, we divided the dataset of species whose sampling sites spanned large geographic breaks in smaller geographic regions. In these cases, models were built considering only locations within the same geographic area. This partitioning was applied to *G. tigrinus*, whose dataset was divided into *G. tigrinus* from its native range in North America and *G. tigrinus* from Europe, and *P. maeoticus*, whose dataset was divided into *P. maeoticus* from the Black Sea and *P. maeoticus* from the Caspian Sea. The exception to this procedure was *G. oceanicus,* that despite having sampling locations from both sides of the Atlantic Ocean, also had one from Iceland. We assumed Iceland could act as stepping stone across continents and therefore complying with isolation‐by‐distance expectations of continuous populations. All statistics and data plotting were conducted in R.3.2.3 software (R Development Core Team, 2011).

## 3 RESULTS

### 3.1 Indices of genetic diversity across species and between invasive and non‐invasive species

A total of 784 sequences were used for seven species, which represented 59 populations (Table S1). The size of workable mtDNA‐COI fragment varied among species from 605 bp for *G. salinus* to 490 bp for *G. oceanicus* (Table 1). The highest number of segregation sites of all sampled locations was observed in the *P. maeoticus* population from Astara (*S*_talesh_ = 63), while the lowest was *S* = 0 observed in several populations of *G. tigrinus* and *G. oceanicus*. The highest number of haplotypes within a sampling location was detected in the *G. locusta* population from Falckenstein (*nH*_Falkenstein_ = 22), and the lowest was the several nHap = 1 associated with the locations that had *S* = 0. Haplotype diversity, as a standardized measure of genetic diversity, showed much more homogeneous distribution with Hd = 1 observed in four populations of *P. maeoticus* and Hd = 0 than those populations constituted by a single haplotype. Lastly, nucleotide diversity had its highest value recorded in the *P. maeoticus* population from Astara (π_Astara_ = 0.048) and lowest was π = 0 associated with the locations where only one haplotype was found. The complete set of diversity indices is shown in Table 1. Statistical analyses revealed no significant differences between the number of segregation sites, haplotype diversity, and nucleotide diversity within native range of both invasive and non‐invasive species (Mann–Whitney–Wilcoxon, *S*:* W*_invasive vs. non‐invasive_ = 214, *p* = .970; Hd: *W*_invasive vs. non‐invasive_ = 271.5, *p* = .166; π: *W*_invasive vs. non‐invasive_ = 90.5, *p* = .307). Phylogenetic reconstructions revealed branch support above 75% bootstrap value in line with previous studies that revealed the existence of highly divergent evolutionary lineages (Figure. S1). NCBI accession numbers are available in Table S1.

**Table 1:** Indices of genetic diversity calculated for each population within each species. Alignment and trimming of the sequences were performed independently for each species. The species‐specific total size of COI fragment is shown in the respective header

Population	*n*	*S*	*n*Hap	Hd	π	Distribution
*G. locusta*—570 bp						
Falckenstein	28	35	22	0.986	0.005	Native
Helgoland	24	23	11	0.862	0.006	Native
Warnemünde	18	21	13	0.954	0.005	Native
*G. salinus*—605 bp						
Falckenstein	11	13	7	0.873	0.007	Native
Helgoland	15	26	6	0.762	0.010	Native
Travemünde	14	23	9	0.835	0.012	Native
Puck Bay	7	5	4	0.714	0.003	Native
*G. tigrinus*—509 bp						
Travemünde	10	0	1	0.000	0.000	Non‐native
Liu	22	20	5	0.732	0.018	Non‐native
Pärnu	19	22	7	0.784	0.017	Non‐native
St.John	9	1	2	0.222	0.000	Non‐native
St.Lawrence	24	11	2	0.290	0.006	Non‐native
Huron	7	0	1	0.000	0.000	Native
Berry creek	11	2	3	0.655	0.002	Native
Delaware	6	11	3	0.600	0.007	Native
Deemers Beach	19	8	2	0.491	0.008	Native
Virginia	40	25	18	0.918	0.011	Native
Hudson	25	1	2	0.080	0.000	Non‐native
Rhode Island	10	4	5	0.756	0.002	Native
Chesapeake	12	5	2	0.409	0.004	Native
Neuse	9	3	4	0.583	0.002	Non‐native
Turku	10	21	4	0.711	0.021	Non‐native
Vistula	10	19	2	0.200	0.007	Non‐native
Brody	9	21	4	0.806	0.020	Non‐native
Byton	9	20	3	0.722	0.022	Non‐native
Anleger	10	0	1	0.000	0.000	Non‐native
Dierhagen	10	20	3	0.733	0.021	Non‐native
Ruhr Metropolis	6	4	3	0.800	0.004	Non‐native
Werra	10	0	1	0.000	0.000	Non‐native
Gouwzee	10	5	2	0.200	0.002	Non‐native
Bann	9	9	3	0.556	0.007	Non‐native
Neagh	12	4	3	0.530	0.003	Non‐native
*G. oceanicus*—490 bp						
Geomar	14	2	2	0.143	0.001	Native
Maine	12	3	3	0.621	0.003	Native
Maine2	21	0	1	0.000	0.000	Native
St.Lawrence	17	2	3	0.485	0.002	Native
Sudurland	8	0	1	0.000	0.000	Native
Poland	42	11	8	0.347	0.003	Native
*P. maeoticus*—515 bp						
Bandar‐e Anzali	29	6	6	0.374	0.001	Native
Jafrud	22	6	6	0.411	0.001	Native
Shafarud	22	13	11	0.714	0.003	Native
Sulina1	7	7	7	1.000	0.005	Native
Sulina2	8	6	6	0.929	0.003	Native
Kazantip	5	16	5	1.000	0.016	Native
Astara	9	63	9	1.000	0.048	Native
Talesh	8	56	7	0.964	0.029	Native
Gisoom	6	6	5	0.933	0.004	Native
Bandar‐e Anzali2	7	56	6	0.952	0.031	Native
Kia	6	5	5	0.933	0.003	Native
Motel	6	6	6	1.000	0.004	Native
Noor	6	4	5	0.933	0.003	Native
Mahmood	8	7	8	1.000	0.003	Native
Khazar	8	53	6	0.893	0.027	Native
*G. zaddachi*—588 bp						
Warnemünde	24	44	10	0.667	0.00692	Native
Kronenloch	26	46	12	0.926	0.01542	Native
United Kingdom	5	4	2	0.4	0.00272	Native
*O. crassus*—597bp						
Gisom	14	6	3	0.538	0.00392	Native
Havigh	18	22	8	0.778	0.00858	Native
Chaboksar	9	4	2	0.389	0.00274	Native

Diversity indices abbreviations stand as following: *n* = number of individual analyzed, *n*Hap = number of haplotypes, Hd = haplotype diversity, *S* = segregation sites, π = nucleotide diversity.

### 3.2 Population differentiation and evolutionary divergence across and between invasive and non‐invasive species

Pairwise *F*_ST_ comparisons reported a wide range of values across species, as well as percentage of statistically significant comparisons that was evaluated for *p*‐values < .01 (Narum, 2006). In *G. locusta*, pairwise values ranged between 0.015 and 0.414, and 66% of pairwise comparisons were significant. For *G. salinus*, pairwise values ranged between 0 and 0.604, and 83% of the total comparisons were significant. In the case of *G. tigrinus*, pairwise *F*_ST_ ranged from 0 and 1.000 with 78% of the total comparisons being significant. All comparisons were significant in the case of *G. oceanicus*, where the *F*_ST_ ranged from 0.422 and 1.000. For *G. zaddachi*, the *F*_ST_ ranged from 0.173 and 0.236, and 66% of the comparisons were significant. In the case of *P. maeoticus*, estimates ranged between 0 and 0.968, with only 36% being significant. None of the pairwise comparisons performed among *O. crassus* sampling locations was significant (Figures S2a–S2f). Average estimates of evolutionary distances (*d*) produced a wide range of values across species. Briefly, *d* ranged between 0.006 and 0.011 for *G. locusta*, 0.010 and 0.019 for *G salinus*, 0.000 and 0.095 for *G. tigrinus*, 0.003 and 0.027 for *G. oceanicus*, 0.008 and 0.015 for *G. zaddachi*, 0.001 and 0.117 for *P. maeoticus*, and 0.005 and 0.008 for *O. crassus* (Tables S2–S8).

**Figure 1:**
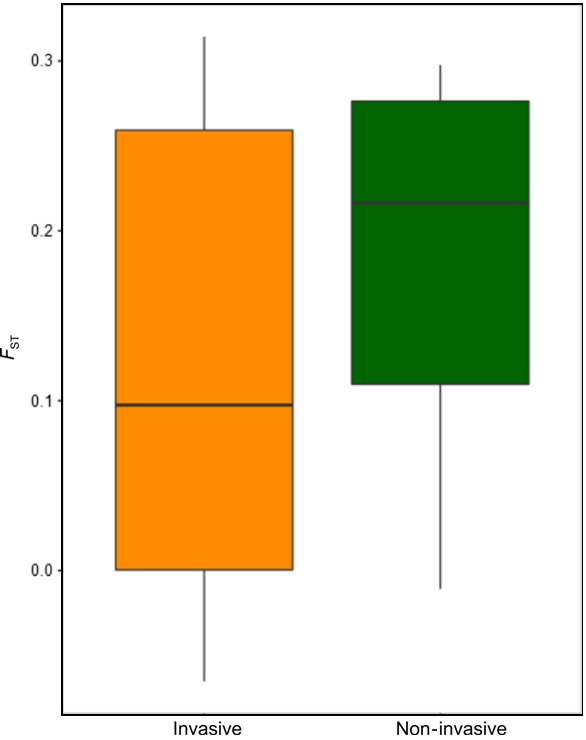
Average *F*_ST_ between invasive and non‐invasive species. Visual representation of the average and standard deviation calculated from pairwise *F*_ST_ estimates of each species group. *Native* refers to species that remain strictly in their native range, while *invasive* are those that have shown capacity to colonize or expand its range after introduction. The status *native* included *G. locusta*,* G. salinus*,* G. Oceanicus,* and *G. zaddachi*. The group invasive included *G. tigrinus*,* P. maeoticus,* and *O. crassus*

**Figure 2:**
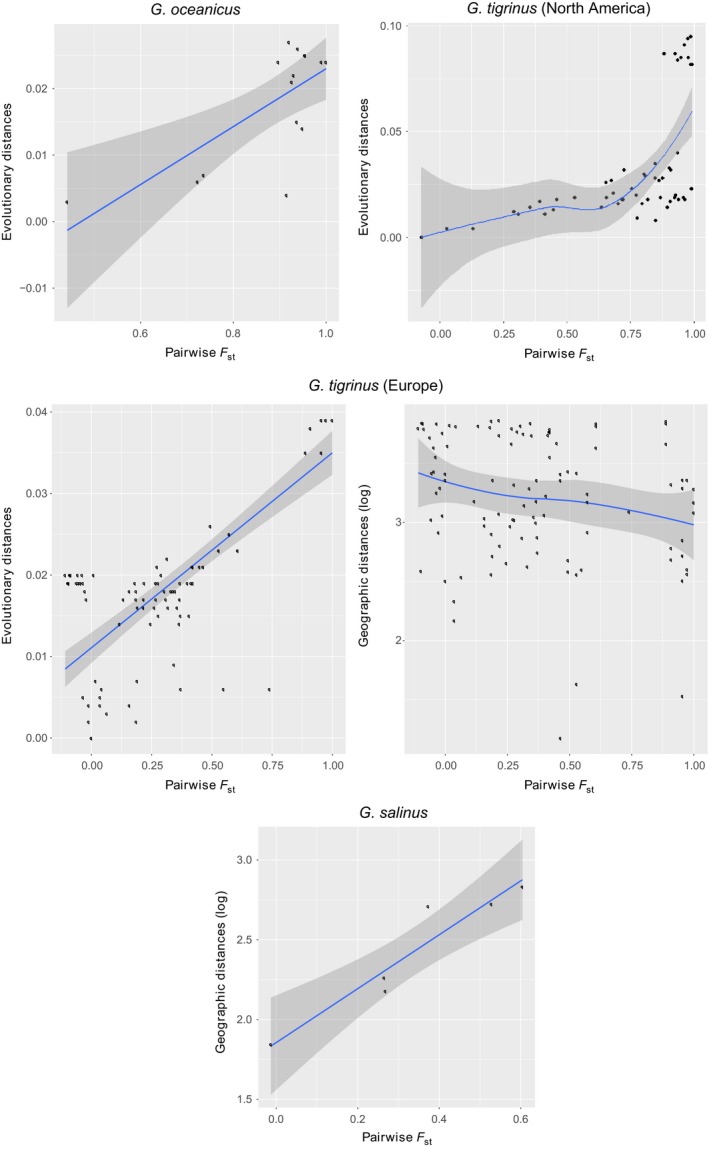
Visual representation of the statistically significant relationships inferred with linear models. Linear relationships were estimated and tested according to the following formula: *F*_ST_ ~ evolutionary distance + geographic distance for each species. The *x*‐axis represents population differentiation while the *y*‐axis depicts the variable or variables that were found to relate *x*‐axis variation

The *F*_ST_ average obtained among the populations of non‐invasive species was significantly higher than the *F*_ST_ average obtained among populations of invasive species in their native range (Mann–Whitney–Wilcox: average *F*_ST non‐invasive_ = 0.019, average *F*_ST_ _invasive_ = 0.011, *W* = −4.038, *p* = .002) (Figure 1). Linear models were built for all species whose dataset provided enough points to comply with statistical computation; therefore, the relationship between *F*_ST_ and evolutionary divergence plus geographic distances was not performed for *G. locusta*,* G. zaddachi,* and *O. crassus*. Evolutionary distances alone explained the population differentiation found among the native locations of *G. tigrinus* in North America and *G. oceanicus* (*G. tigrinus*_North America_: *t* = 4.287, *p* = .00; *G. oceanicus*:* t* = 3.305, *p* = .006). Linear models explained only 33% and 43% of *F*_ST_ variation for *G. tigrinus*_North America_ (*R* ^2^ = .33, *p* < .001) and *G. oceanicus*: (*R* ^2^ = .43, *p* = .013), respectively (Figure 2; Table 2). For *G. salinus*, we found a pattern suggestive of isolation by distance, where higher *F*_ST_ were explained by larger geographic distances (*G. salinus*:* t* = 4.603, *p* = .019, *R* ^2^ = .87, *p* = .019) (Figure 2; Table 2). Interestingly, we found that the pairwise *F*_ST_ obtained among the locations of *G. tigrinus* (Europe) positively correlated with evolutionary distances (*G. tigrinus*_Europe_: *t* = 12.847, *p* < .001) but negatively with geographic distance (*G. tigrinus*_Europe_: *t* = ‐2.916, *p* = .004) in the model that explained 68% of *F*_ST_ variation (*R* ^2^ = .68, *p* < .001) (Figure 2; Table 2). The model with *P. maeoticus* (Caspian Sea) was not significant.

**Table 2:** Summarized statistics obtained from the linear models. The relationship between population differentiation (average *F*_ST_) with both evolutionary and geographic distances was estimated based on the formula average *F*_ST_ ~ evolutionary distance + geographic distance

	Estimate	*SE*	*t*	*p*
*G. salinus*				
Intercept	−0.999	0.219	−4.556	**.020**
Evolutionary distance	11.769	12.860	0.915	.428
Geographic distance	0.484	0.105	4.603	**.019**
*G. tigrinus* (North America)				
Intercept	0.906	0.146	6.171	**.000**
Evolutionary distance	4.287	0.985	4.348	**.000**
Geographic distance	−0.085	0.046	−1.858	.070
*G. tigrinus* (Europe)				
Intercept	0.213	0.127	1.679	.096
Evolutionary distance	25.007	1.947	12.847	**2e−16**
Geographic distance	−0.107	0.037	−2.916	**.004**
*G. oceanicus*				
Intercept	0.753	0.244	3.081	**.010**
Evolutionary distance	11.423	3.457	3.305	**.006**
Geographic distance	−0.022	0.065	−0.346	.735

bold marked values refer to statistically significant effects

## 4 DISCUSSION

The distribution of neutral genetic diversity provides important clues to understand the processes and mechanisms underlying biological invasions at the molecular level. Our study showed that despite the wide variation observed in indices of genetic diversity within each species in their native ranges, no significant differences were observed at any level between populations of non‐invasive and invasive species. Population genetic structure was pervasive among pairwise comparisons within each species, but interestingly, populations of non‐invasive species produce significantly higher levels of differentiation than those of invasive in their native range. We also observed the occurrence of deep evolutionary lineages for almost all species, a feature that is commonly found among gammarids and documented in a series of related studies (Cristescu et al., 2003; Hou et al., 2014; Kelly, MacIsaac, et al., 2006). The relationships between population differentiation, geographic distances, and evolutionary distances revealed a distinct sort of patterns. However, only those observed in *G. salinus* and *G. tigrinus* in Europe did fall in line with our expectations.

### 4.1 Genetic diversity and population differentiation of non‐invasive species

Of all non‐invasive species analyzed in our study, *G. oceanicus* distribution covers the widest geographic area. Populations of this species presented moderate‐to‐low levels of genetic diversity both at summary statistics and *d* estimates. Krebes, Blank, and Bastrop (2011) characterized a phylogeographic pattern dominated by divergent lineages confined to specific geographic regions as a product of Quaternary glaciations, with the current distribution being a result of natural range expansions following the last glacial Maximum (LGM). Similarly, deep lineage divergence was also observed for *G. locusta* in this study, which is consistent to those reported by Hou, Fu, and Li (2007). Similar congruence of patterns was found in *G. zaddachi* and G. *salinus,* as suggested by high variation in the number of segregation sites. Overall, differentiation estimates showed a variable range from one species to another which perhaps reflects evolutionary history of each species. The wide area inhabited by each species certainly favored the evolution of distinct populations. *Gammarus locusta*,* G. salinus,* and G. *zaddachi* presented less structure, with punctual cases that could be justified by the geographic specificities of the environment. For instances, *F*_ST_ estimates of *G. locusta* revealed that the population from Falckenstein is isolated from the others, which can be explained by Falckenstein being located in an inner location within a fjord that extends kilometers into continent. The other two populations are most likely connected in the Baltic Sea. Regarding *G. zaddachi,* results suggest a phylogeographic break between North and Baltic Sea, as previously observed by Bulnheim and Scholl (1981). This observation is supported by the high and significant *F*_ST_ values between Baltic and UK populations, and absence of differentiation among those within the Baltic Sea. *Gammarus salinus* presented a contrasting pattern; *F*_ST_ values suggested the existence of highly differentiating populations within the Baltic Sea. This could be a result of *G. salinus* remaining confined to coastal pockets or brackish periglacial refugia and expanded after LGM (Hewitt, 2000; Maggs et al., 2008) or due to local adaptation restraining the gene flow and leading to the evolution of distinct *G. salinus* populations over evolutionary time scales (Via, 1999; Via 2001). Nevertheless, our study provided the first information at population genetic level for *G. salinus* that can be a valuable resource for cataloging biodiversity of the Baltic Sea at the molecular level, which is suspected to be significantly reduced in comparison with other regions (Johannesson & Andre, 2006).

### 4.2 Genetic diversity and population differentiation of invasive species

Of all invasive species investigated in our study, *G. tigrinus* was one of the two species where published information partially overlapped with ours and in this case much due to the work of Kelly, MacIsaac, et al. (2006), Kelly, Muirhead, et al. (2006). Those authors identified four main clades—N1, N2, N3, and N4—across the species distribution range. Genetic differentiation (*F*_ST_) and evolutionary distances estimates (*d*) among the Baltic Sea locations in our study suggest that (1) Travemünde is dominated by a single haplotype and is a very likely representative of clade N1, which is present in northern Europe, and (2) Pärnu and Liu are similar to populations composed by clades N1 and N4 (also present in Europe). Travemünde population is apparently composed of descendents of *G. tigrinus* introduced in the Werra river in the 1960s, while the other locations suggest a stepwise introduction along the pathway North America–British Isles–Baltic Sea (Kelly, Muirhead, et al., 2006). On the one hand, comparison of average genetic diversity indices between populations in the native and non‐native ranges revealed no significant differences in the number of segregation (*S*) sites and haplotype diversity (Hd). This is not surprising, as Kelly, MacIsaac, et al. (2006), Kelly, Muirhead, et al. (2006) also reported contrasting patterns between populations in the native and non‐native ranges when performing pairwise comparisons between sources and sinks (Kelly, Muirhead, et al., 2006). In contrast, the significantly higher average nucleotide diversity (π) among non‐native populations of *G. tigrinus* in this study might be attributed to the introduction of highly variable populations of Pärnu and Liu. Information on evolutionary history of *P. maeoticus* was readily available through the work of Nahavandi, Ketmaier, Plath, and Tiedemann (2013). In our study, we added three more populations from the Caspian region and confirmed previously observed existence of divergent clades within the Caspian Sea (Nahavandi et al., 2013)—suggested by high variance in segregation sites and haplotype diversity in native range. We were not able to identify the distinct Black Sea clade though, which can be justified by the fact that not all of the locations sequenced by Nahavandi et al. (2013) were used in this study because they fell short in the number of individuals. Nevertheless, pairwise *F*_ST_ values among the newly added populations of Bandar‐e Anzali, Jafrud, and Shafarud fell into the range of estimates of those obtained by Nahavandi et al. (2013). *Obesogammarus crassus* is another Ponto‐Caspian species that is gradually extending its range northward. Diversity indices did not find evidence that suggested existence of deeply divergent lineages at least at the extent of those reported in *P. maeoticus*. Still, the lack of deeply divergent lineages—a common trait among amphipods—can be explained by the fact that we did not sample the Black Sea, where the phylogeographic break is usually detected in Ponto‐Caspian fauna (Cristescu et al., 2003).

### 4.3 Genetic diversity and population differentiation between invasive and non‐invasive species

We also tested a possible relationship between genetic diversity in the native range and invasion propensity. Our results indicated that there was no difference between the average diversity estimates obtained for any group. Still, we found that the degree of differentiation among the populations of non‐invasive species is higher than that of invasive species in their native region. Joint interpretation of the comparisons of diversity and differentiation suggests that genetic diversity is more segregated in non‐invasive species. Perhaps the most parsimonious justification is that this conjugation of patterns constitutes a spurious correlation between distinct evolutionary histories that shaped the variation and distribution of genetic diversity independently in each species and propensity to invade. Still, evidence obtained at molecular level suggests that the nature of genetic variation is more important in establishment and invading success than the overall quantity (Dlugosch et al. 2015). It is clear though, that the resolution obtained by screening diversity at a single genetic marker does not provide the necessary amount of information to perform in‐depth analyses regarding causality. Therefore, potential causal relationships fall in the realm of speculation. Noteworthy, the observation that non‐invasive species present higher levels of population differentiation than that of invasive ones in their native range is in line with Gaither et al. (2013). Gaither and colleagues reported a negative correlation between dispersal—as the likelihood to achieve non‐native ranges—and *F*_ST_ at native ranges, further suggesting that non‐invasive species have their populations more structured (Gaither et al., 2013). An alternative explanation would be that less differentiation, as a result of higher migration among populations, would facilitate the spread of mutations with no fitness value in the native range, but advantageous in the introduced range (Morjan & Rieseberg, 2004; Slatkin, 1987). Because the species analyzed in our study have similar life histories, one could further hypothesize that the variable invasion success observed among gammarids could also be linked to the likelihood of the right genotype being “picked” by anthropogenic mechanisms from the pool available in native range and transported to non‐native locations. Considering the “right genotype” to have evolved somewhere in the native range, the probability of picking it up when sampling a random population is directly proportional to the gene flow among populations.

### 4.4 Relationships between estimates of population differentiation, geographic, and evolutionary distances

The high degree of divergence often reported among members of this superfamily led us to investigate relationships between population differentiation and distinct distance measures. We investigated mostly relationships in native populations; the exception was *G. tigrinus* from which we were able to analyze relationships both for its North American native range and European non‐native range. For those distributed in their native ranges, we found distinct patterns of differentiation–distances relationships (Figure 2). *Gammarus salinus* was the only species for which we found a positive correlation between population differentiation and geographic distances (Table 2). This pattern can be justified by the coastal habitat occupied by *G. salinus*, which constrains dispersal among locations and permits the evolution and maintenance of site‐specific genetic diversity (Gaston & Spicer, 2001). However, we cannot exclude the potential effect of local adaptation to each site that could lead to gene flow restrictions among populations (Orsini, Vanoverbeke, Swillen, Mergeay, & Meester, 2013). Difference between neutral and selective drivers behind the isolation pattern identified here would therefore require the identification of the possible selective pressures and stronger statistical approaches to discriminate which of the two better explains population differentiation (Meirmans, 2015).

Interestingly, none of the species reported positive linear relationship with geographic distances; *G. oceanicus* and *G. tigrinus* in its American native range revealed a positive relationship between differentiation and evolutionary distances instead, while the geographic variable attained no significant weight (Table 2). This indicates that different lineage composition drives the differentiation among populations of *G. tigrinus* and *G. oceanicus,* and despite suggestive of lineages being locally adapted, no empirical evidence exists to support such claim. Mitochondrial variation shaped by selective processes other than strong purifying selection is not commonly reported in studies of natural populations, but see Silva, Lima, Martel, and Castilho (2014) for an evidence of thermal adaptation of anchovies linked to variation in mitochondrial cytochrome *b* (Silva et al., 2014).

The absence of any relationship between the variables explored here and differentiation among *P. maeoticus* can be tentatively explained by a mix of ancient and contemporary factors. Alternatively, it is possible that Quaternary glaciation cycles have impacted the distribution of the species within and between the basins of the Caspian and Black Sea and shaped a genetic patchiness that relates neither with geographic or evolutionary distances (Hewitt, 1996). However, intense ship traffic between basins, that started after the completion of the Don‐Volga canal in the 1950s, and other unintentional translocation activities might have disturbed natural distribution patterns (Audzijonyte, Wittmann, Ovcarenko, & Väinölä, 2009; Grigorovich, Therriault, & MacIsaac, 2003).

Particularly interesting are the relationships between the three variables for the European non‐native distribution of *G. tigrinus*, where population differentiation negatively correlates with geographic distances (Figure 2; Table 2), but positively with evolutionary distance. These apparently contrasting patterns are partially in line with the introduction history of *G. tigrinus* in Europe, because the introduction has occurred at limited spatial scales and from multiple introduction events (Kelly, Muirhead, et al., 2006). This might have originated highly structured populations within the new range immediately after the introduction, therefore explaining the negative correlation between differentiation and spatial distances. Next, heterogeneous patterns of dispersal among lineages, where some lineages show tendency to disperse more than others, would be a possible justification for the positive relationship observed between geographic and evolutionary distances. Other explanations may be gene surfing, a phenomena of random causes that might occur in expanding populations and promotes structure and diversification (Excoffier & Ray, 2008) if different dispersal abilities are encoded in each lineage or the sorting effect of natural selection in the sink populations. The apparent niche specialization observed in the Baltic Sea for *G. tigrinus* could indicate an effect of natural selection in the invaded area (Herkül et al., 2016).

### 4.5 Future direction and caveats of the study

The mtDNA‐COI was used because it is the most represented gene in public databases and allowed us to cover a high number of species and populations. The drawback is that we had to standardize fragment lengths among recorded sequences for each species, in order to avoid creating intraspecific artificial variation. However, the substantial level of polymorphism observed at a gene known to be as conservative as mtDNA‐COI suggests that rather than extending COI representation, a much higher number of genetic markers and extensive sampling are required to validate and understand the patterns brought in by our work. This would offer the possibility to obtain better estimates of population differentiation, as those are dependent on well‐characterized within‐population diversity to produce robust conclusions (Meirmans & Hedrick, 2011). Clearly, interpreting the genetic signatures imprinted in the genome of these species would be much facilitated by genome‐widescreen. Also, we did not take into account any contemporary demographic process nor did we make any deep inferences of signatures of contemporary demographic events. Phylogenetic studies mentioned throughout this manuscript strongly support rapid diversification and expansion among gammarids occurring as far back as the Cretaceous.

Another important point is that species presented as non‐invasive in our study are assigned to be non‐invasive as they have not been reported in areas outside their native ranges. However, morphological identification of gammarids to the species level can be a challenging task and a species might also invade in new areas in the future; therefore, we acknowledge the possibility of one or more of currently assigned non‐invasive species becoming invasive in the future.

## CONFLICT OF INTEREST

None declared.

## References

[ece33208-bib-0001] Audzijonyte, A. , Wittmann, K. J. , Ovcarenko, I. , & Väinölä, R. (2009). Invasion phylogeography of the Ponto‐Caspian crustacean Limnomysis benedeni dispersing across Europe. Diversity and Distributions, 15, 346–355.

[ece33208-bib-0002] Bij de Vaate, A. , Jazdzewski, K. , Ketelaars, H. A. , Gollasch, S. , & Van der Velde, G. (2002). Geographical patterns in range extension of Ponto‐Caspian macroinvertebrate species in Europe. Canadian Journal of Fisheries and Aquatic Sciences, 59, 1159–1174.

[ece33208-bib-0003] Bock, D. G. , Caseys, C. , Cousens, R. D. , Hahn, M. A. , Heredia, S. M. , Hübner, S. , … Rieseberg, L. H. (2015). What we still don't know about invasion genetics. Molecular Ecology, 24, 2277–2297. 25474505 10.1111/mec.13032

[ece33208-bib-0004] Briski, E. , Allinger, L. E. , Balcer, M. , Cangelosi, A. , Fanberg, L. , Markee, T. P. , … Reavie, E. D. (2013). Multidimensional approach to invasive species prevention. Environmental Science & Technology, 47, 1216–1221. 23293915 10.1021/es3029445

[ece33208-bib-0005] Briski, E. , Chan, F. T. , MacIsaac, H. J. , & Bailey, S. A. (2014). A conceptual model of community dynamics during the transport stage of the invasion process: A case study of ships’ ballast. Diversity and Distributions, 20, 236–244.

[ece33208-bib-0006] Bulnheim, H.‐P. , & Scholl, A. (1981). Genetic variation between geographic populations of the amphipods *Gammarus zaddachi* and *G. salinus* . Marine Biology, 64, 105–115.

[ece33208-bib-0007] Capinha, C. , Essl, F. , Seebens, H. , Moser, D. , & Pereira, H. M. (2015). The dispersal of alien species redefines biogeography in the Anthropocene. Science, 348, 1248–1251. 26068851 10.1126/science.aaa8913

[ece33208-bib-0008] Casties, I. , Seebens, H. , & Briski, E. (2016). Importance of geographic origin for invasion success: A case study of the North and Baltic Seas versus the Great Lakes–St. Lawrence River region. Ecology and Evolution, 6, 8318–8329. 27878098 10.1002/ece3.2528 PMC5108280

[ece33208-bib-0501] Colautti, R. I. , & Lau, J. A. (2015). Contemporary evolution during invasion: Evidence for differentiation, natural selection, and local adaptation. Molecular Ecology, 24, 1999–2017. 25891044 10.1111/mec.13162

[ece33208-bib-0009] Costa, F. , Henzler, C. , Lunt, D. , Whiteley, N. , & Rock, J. (2009). Probing marine *Gammarus* (Amphipoda) taxonomy with DNA barcodes. Systematics and Biodiversity, 7, 365–379.

[ece33208-bib-0010] Cristescu, M. E. (2015). Genetic reconstructions of invasion history. Molecular Ecology, 24, 2212–2225. 25703061 10.1111/mec.13117

[ece33208-bib-0011] Cristescu, M. E. , Hebert, P. D. , & Onciu, T. M. (2003). Phylogeography of Ponto‐Caspian crustaceans: A benthic–planktonic comparison. Molecular Ecology, 12, 985–996. 12753217 10.1046/j.1365-294x.2003.01801.x

[ece33208-bib-0502] Dlugosch, K. M. , Anderson, S. R. , Braasch, J. , Cang, F. A. , & Gillette, H. D. (2015). The devil is in the details: Genetic variation in introduced populations and its contributions to invasion. Molecular Ecology, 24, 2095–2111. 25846825 10.1111/mec.13183

[ece33208-bib-0012] DAISIE European Invasive Alien Species Gateway. Retrieved from http://www.europe-aliens.org

[ece33208-bib-0013] Edgar, R. C. (2004). MUSCLE: Multiple sequence alignment with high accuracy and high throughput. Nucleic Acids Research, 32, 1792–1797. 15034147 10.1093/nar/gkh340 PMC390337

[ece33208-bib-0014] Estoup, A. , & Guillemaud, T. (2010). Reconstructing routes of invasion using genetic data: Why, how and so what? Molecular Ecology, 19, 4113–4130. 20723048 10.1111/j.1365-294X.2010.04773.x

[ece33208-bib-0015] Ewing, G. B. , & Jensen, J. D. (2016). The consequences of not accounting for background selection in demographic inference. Molecular Ecology, 25, 135–141. 26394805 10.1111/mec.13390

[ece33208-bib-0016] Excoffier, L. , & Lischer, H. E. L. (2009). Arlequin suite ver 3.5: A new series of programs to perform population genetics analyses under Linux and Windows. Molecular Ecology Resources, 10, 564–567. 10.1111/j.1755-0998.2010.02847.x 21565059

[ece33208-bib-0017] Excoffier, L. , & Ray, N. (2008). Surfing during population expansions promotes genetic revolutions and structuration. Trends in Ecology & Evolution, 23, 347–351. 18502536 10.1016/j.tree.2008.04.004

[ece33208-bib-0018] Gaither, M. R. , Bowen, B. W. , & Toonen, R. J. (2013). Population structure in the native range predicts the spread of introduced marine species. Proceedings of the Royal Society B: Biological Sciences, 280, 20130409. 23595272 10.1098/rspb.2013.0409 PMC3652461

[ece33208-bib-0019] Gaston, K. J. , & Spicer, J. I. (2001). The relationship between range size and niche breadth: A test using five species of *Gammarus* (Amphipoda). Global Ecology and Biogeography, 10, 179–188.

[ece33208-bib-0020] GISD (2017). Global Invasive Species Database. Retrieved from http://www.iucngisd.org/gisd/

[ece33208-bib-0021] Grigorovich, I. A. , Therriault, T. W. , & MacIsaac, H. J. (2003). History of aquatic invertebrate invasions in the Caspian sea In Pederson Judith (Ed.), Marine bioinvasions: Patterns, processes and perspectives (pp. 103–115), Springer Netherlands: Springer.

[ece33208-bib-0022] Hall, T. A. (1999). BioEdit: A user‐friendly biological sequence alignment editor and analysis program for Windows 95/98/NT. Nucleic acids symposium series, 41, 95–98.

[ece33208-bib-0023] Hellmann, J. J. , Byers, J. E. , Bierwagen, B. G. , & Dukes, J. S. (2008). Five potential consequences of climate change for invasive species. Conservation Biology, 22, 534–543. 18577082 10.1111/j.1523-1739.2008.00951.x

[ece33208-bib-0024] Herkül, K. , Lauringson, V. , & Kotta, J. (2016). Specialization among amphipods: The invasive *Gammarus tigrinus* has narrower niche space compared to native gammarids. Ecosphere, 7, e01306.

[ece33208-bib-0025] Hewitt, G. M. (1996). Some genetic consequences of ice ages, and their role in divergence and speciation. Biological Journal of the Linnean Society, 58, 247–276.

[ece33208-bib-0026] Hewitt, G. (2000). The genetic legacy of the Quaternary ice ages. Nature, 405, 907–913. 10879524 10.1038/35016000

[ece33208-bib-0027] Holsinger, K. E. , & Weir, B. S. (2009). Genetics in geographically structured populations: Defining, estimating and interpreting FST. Nature Reviews Genetics, 10, 639–650. 10.1038/nrg2611 PMC4687486 19687804

[ece33208-bib-0028] Hou, Z. , Fu, J. , & Li, S. (2007). A molecular phylogeny of the genus *Gammarus* (Crustacea: Amphipoda) based on mitochondrial and nuclear gene sequences. Molecular Phylogenetics and Evolution, 45, 596–611. 17686635 10.1016/j.ympev.2007.06.006

[ece33208-bib-0029] Hou, Z. , & Sket, B. (2016). A review of Gammaridae (Crustacea: Amphipoda): The family extent, its evolutionary history, and taxonomic redefinition of genera. Zoological Journal of the Linnean Society, 176, 323–348.

[ece33208-bib-0030] Hou, Z. , Sket, B. , Fišer, C. , & Li, S. (2011). Eocene habitat shift from saline to freshwater promoted Tethyan amphipod diversification. Proceedings of the National Academy of Sciences, 108, 14533–14538. 10.1073/pnas.1104636108 PMC3167504 21844362

[ece33208-bib-0031] Hou, Z. , Sket, B. , & Li, S. (2014). Phylogenetic analyses of Gammaridae crustacean reveal different diversification patterns among sister lineages in the Tethyan region. Cladistics, 30, 352–365. 10.1111/cla.12055 34794244

[ece33208-bib-0032] Johannesson, K. , & Andre, C. (2006). Invited review: Life on the margin: Genetic isolation and diversity loss in a peripheral marine ecosystem, the Baltic Sea. Molecular Ecology, 15, 2013–2029. 16780421 10.1111/j.1365-294X.2006.02919.x

[ece33208-bib-0033] Keller, R. P. , Drake, J. M. , Drew, M. B. , & Lodge, D. M. (2011). Linking environmental conditions and ship movements to estimate invasive species transport across the global shipping network. Diversity and Distributions, 17, 93–102.

[ece33208-bib-0034] Kelly, D. W. , MacIsaac, H. J. , & Heath, D. D. (2006). Vicariance and dispersal effects on phylogeographic structure and speciation in a widespread estuarine invertebrate. Evolution, 60, 257–267. 16610318

[ece33208-bib-0035] Kelly, D. W. , Muirhead, J. R. , Heath, D. D. , & Macisaac, H. J. (2006). Contrasting patterns in genetic diversity following multiple invasions of fresh and brackish waters. Molecular Ecology, 15, 3641–3653. 17032263 10.1111/j.1365-294X.2006.03012.x

[ece33208-bib-0036] Krebes, L. , Blank, M. , & Bastrop, R. (2011). Phylogeography, historical demography and postglacial colonization routes of two amphi‐Atlantic distributed amphipods. Systematics and Biodiversity, 9, 259–273.

[ece33208-bib-0037] Leblois, R. , Rousset, F. , Tikel, D. , Moritz, C. , & Estoup, A. (2000). Absence of evidence for isolation by distance in an expanding cane toad (*Bufo marinus*) population: An individual‐based analysis of microsatellite genotypes. Molecular Ecology, 9, 1905–1909. 11091326 10.1046/j.1365-294x.2000.01091.x

[ece33208-bib-0038] Librado, P. , & Rozas, J. (2009). DnaSP v5: A software for comprehensive analysis of DNA polymorphism data. Bioinformatics, 25, 1451–1452. 19346325 10.1093/bioinformatics/btp187

[ece33208-bib-0039] Lockwood, J. L. , Hoopes, M. F. , & Marchetti, M. P. (2013). Invasion ecology. USA: John Wiley & Sons.

[ece33208-bib-0040] Maggs, C. A. , Castilho, R. , Foltz, D. , Henzler, C. , Jolly, M. T. , Kelly, J. , … Väinölä, R. (2008). Evaluating signatures of glacial refugia for North Atlantic benthic marine taxa. Ecology, 89, S108–S122. 19097488 10.1890/08-0257.1

[ece33208-bib-0041] Marrs, R. A. , Sforza, R. , & Hufbauer, R. A. (2008). When invasion increases population genetic structure: A study with *Centaurea diffusa* . Biological Invasions, 10, 561–572.

[ece33208-bib-0042] Meirmans, P. G. (2015). Seven common mistakes in population genetics and how to avoid them. Molecular Ecology, 24, 3223–3231. 25974103 10.1111/mec.13243

[ece33208-bib-0043] Meirmans, P. G. , & Hedrick, P. W. (2011). Assessing population structure: FST and related measures. Molecular Ecology Resources, 11, 5–18. 21429096 10.1111/j.1755-0998.2010.02927.x

[ece33208-bib-0044] Morjan, C. L. , & Rieseberg, L. H. (2004). How species evolve collectively: Implications of gene flow and selection for the spread of advantageous alleles. Molecular Ecology, 13, 1341–1356. 15140081 10.1111/j.1365-294X.2004.02164.x PMC2600545

[ece33208-bib-0045] Muirhead, J. R. , Gray, D. K. , Kelly, D. W. , Ellis, S. M. , Heath, D. D. , & Macisaac, H. J. (2008). Identifying the source of species invasions: Sampling intensity vs. genetic diversity. Molecular Ecology, 17, 1020–1035. 18261046 10.1111/j.1365-294X.2008.03669.x

[ece33208-bib-0046] Nahavandi, N. , Ketmaier, V. , Plath, M. , & Tiedemann, R. (2013). Diversification of Ponto‐Caspian aquatic fauna: Morphology and molecules retrieve congruent evolutionary relationships in *Pontogammarus maeoticus* (Amphipoda: Pontogammaridae). Molecular Phylogenetics and Evolution, 69, 1063–1076. 23764337 10.1016/j.ympev.2013.05.021

[ece33208-bib-0047] Narum, S. R. (2006). Beyond Bonferroni: Less conservative analyses for conservation genetics. Conservation Genetics, 7, 783–787.

[ece33208-bib-0503] Nielsen, R. , & Slatkin, M. (2013). An introduction to population genetics: Theory and applications. Sunderland, MA: Sinauer Associates.

[ece33208-bib-0048] Orsini, L. , Vanoverbeke, J. , Swillen, I. , Mergeay, J. , & Meester, L. (2013). Drivers of population genetic differentiation in the wild: Isolation by dispersal limitation, isolation by adaptation and isolation by colonization. Molecular Ecology, 22, 5983–5999. 24128305 10.1111/mec.12561

[ece33208-bib-0049] Pligin, Y. V. , Matchinskaya, S. , Zheleznyak, N. , & Linchuk, M. (2014). Long‐term distribution of alien species of macroinvertebrates in the ecosystems of the Dnieper Reservoirs. Hydrobiological Journal, 50, 3–17.

[ece33208-bib-0050] Raupach, M. J. , Barco, A. , Steinke, D. , Beermann, J. , Laakmann, S. , Mohrbeck, I. , … Radulovici, A. (2015). The application of DNA barcodes for the identification of marine crustaceans from the North Sea and adjacent regions. PLoS One, 10, e0139421. 26417993 10.1371/journal.pone.0139421 PMC4587929

[ece33208-bib-0051] Ricciardi, A. , & MacIsaac, H. J. (2000). Recent mass invasion of the North American Great Lakes by Ponto–Caspian species. Trends in Ecology & Evolution, 15, 62–65. 10652557 10.1016/s0169-5347(99)01745-0

[ece33208-bib-0052] Roman, J. , & Darling, J. A. (2007). Paradox lost: Genetic diversity and the success of aquatic invasions. Trends in Ecology & Evolution, 22, 454–464. 17673331 10.1016/j.tree.2007.07.002

[ece33208-bib-0053] Saitou, N. , & Nei, M. (1987). The neighbor‐joining method: A new method for reconstructing phylogenetic trees. Molecular Biology and Evolution, 4, 406–425. 3447015 10.1093/oxfordjournals.molbev.a040454

[ece33208-bib-0054] Semenchenko, V. , & Vezhnovetz, V. (2008). Two new invasive Ponto‐Caspian amphipods reached the Pripyat River, Belarus. Aquatic Invasions, 3, 445–447.

[ece33208-bib-0055] Sherman, C. , Lotterhos, K. , Richardson, M. , Tepolt, C. , Rollins, L. , Palumbi, S. , & Miller, A. (2016). What are we missing about marine invasions? Filling in the gaps with evolutionary genomics. Marine Biology, 163, 198.

[ece33208-bib-0056] Silva, G. , Lima, F. P. , Martel, P. , & Castilho, R. (2014). Thermal adaptation and clinal mitochondrial DNA variation of European anchovy. Proceedings of the Royal Society of London B: Biological Sciences, 281, 20141093. 10.1098/rspb.2014.1093 PMC4150322 25143035

[ece33208-bib-0057] Slatkin, M. (1987). Gene flow and the geographic structure of natural populations. Science, 236, 787–793. 3576198 10.1126/science.3576198

[ece33208-bib-0058] Sylvester, F. , Kalaci, O. , Leung, B. , Lacoursière‐Roussel, A. , Murray, C. C. , Choi, F. M. , … MacIsaac, H. J. (2011). Hull fouling as an invasion vector: Can simple models explain a complex problem? Journal of Applied Ecology, 48, 415–423.

[ece33208-bib-0504] R Development Core Team (2011). R: A language and environment for statistical computing. Vienna, Austria: R Foundation for Statistical Computing.

[ece33208-bib-0059] Tamura, K. , & Kumar, S. (2002). Evolutionary distance estimation under heterogeneous substitution pattern among lineages. Molecular Biology and Evolution, 19, 1727–1736. 12270899 10.1093/oxfordjournals.molbev.a003995

[ece33208-bib-0060] Tamura, K. , Stecher, G. , Peterson, D. , Filipski, A. , & Kumar, S. (2013). MEGA6: Molecular evolutionary genetics analysis version 6.0. Molecular Biology and Evolution, 30, 2725–2729. 24132122 10.1093/molbev/mst197 PMC3840312

[ece33208-bib-0505] Via, S. (2001). Sympatric speciation in animals: The ugly duckling grows up. Trends in Ecology & Evolution, 16, 381–390. 11403871 10.1016/s0169-5347(01)02188-7

[ece33208-bib-0061] Via, S. (1999). Reproductive isolation between sympatric races of pea aphids. I. Gene flow restriction and habitat choice. Evolution, 1, 1446–1457. 10.1111/j.1558-5646.1999.tb05409.x 28565574

[ece33208-bib-0062] Vrijenhoek, R. (1994). DNA primers for amplification of mitochondrial cytochrome c oxidase subunit I from diverse metazoan invertebrates. Molecular Marine Biology and Biotechnology, 3, 294–299. 7881515

[ece33208-bib-0063] Wright, S. (1943). Isolation by distance. Genetics, 28, 114. 17247074 10.1093/genetics/28.2.114 PMC1209196

[ece33208-bib-0064] Zhan, A. , Darling, J. A. , Bock, D. G. , Lacoursière‐Roussel, A. , MacIsaac, H. J. , & Cristescu, M. E. (2012). Complex genetic patterns in closely related colonizing invasive species. Ecology and Evolution, 2, 1331–1346. 22957143 10.1002/ece3.258 PMC3434944

