# Supplementary figures and images for: Diversity and distribution of genetic variation in gammarids: Comparing patterns between invasive and non‐invasive species
Source: Ecol Evol. 2017 Aug 22;7(19):7687–98. doi: 10.1002/ece3.3208 (PMC5632605; doi:10.1002/ece3.3208)

*G locusta*

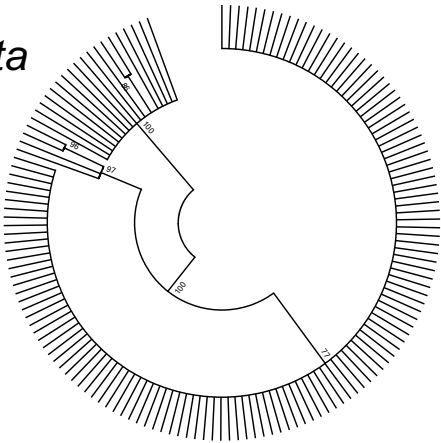

*G salinus*

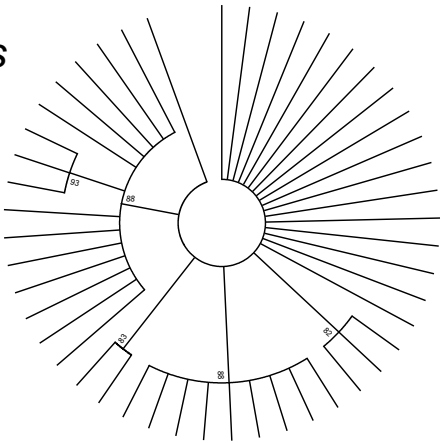

*G tigrinus*

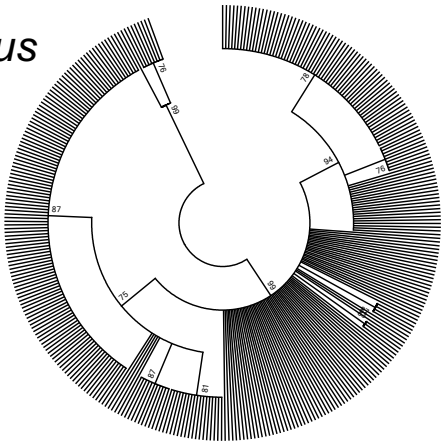

*G oceanicus*

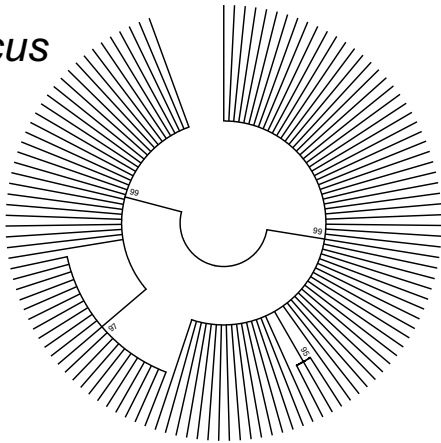

*P meioticus*

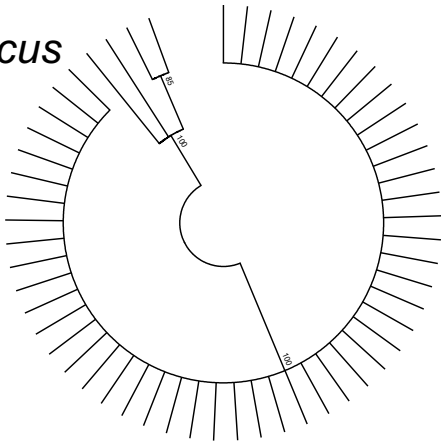

*G zaddachi*

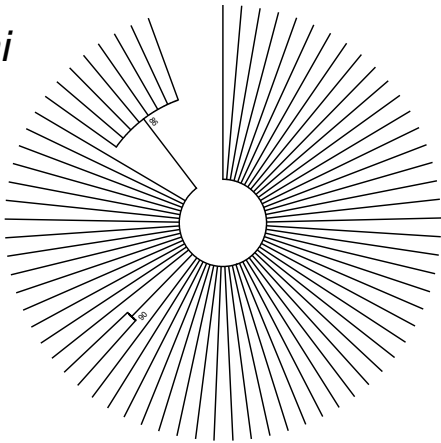

*O crassus*

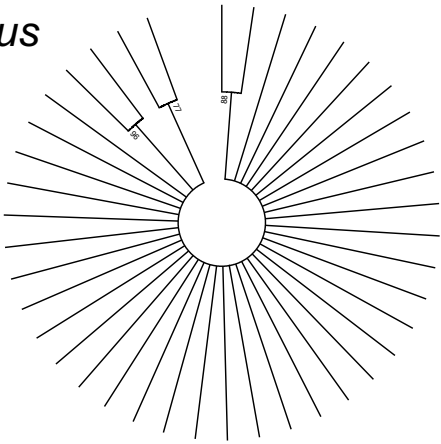

Supplement: Supplementary file 1 [file ECE3-7-7687-s001.pdf]

a)

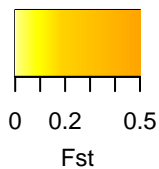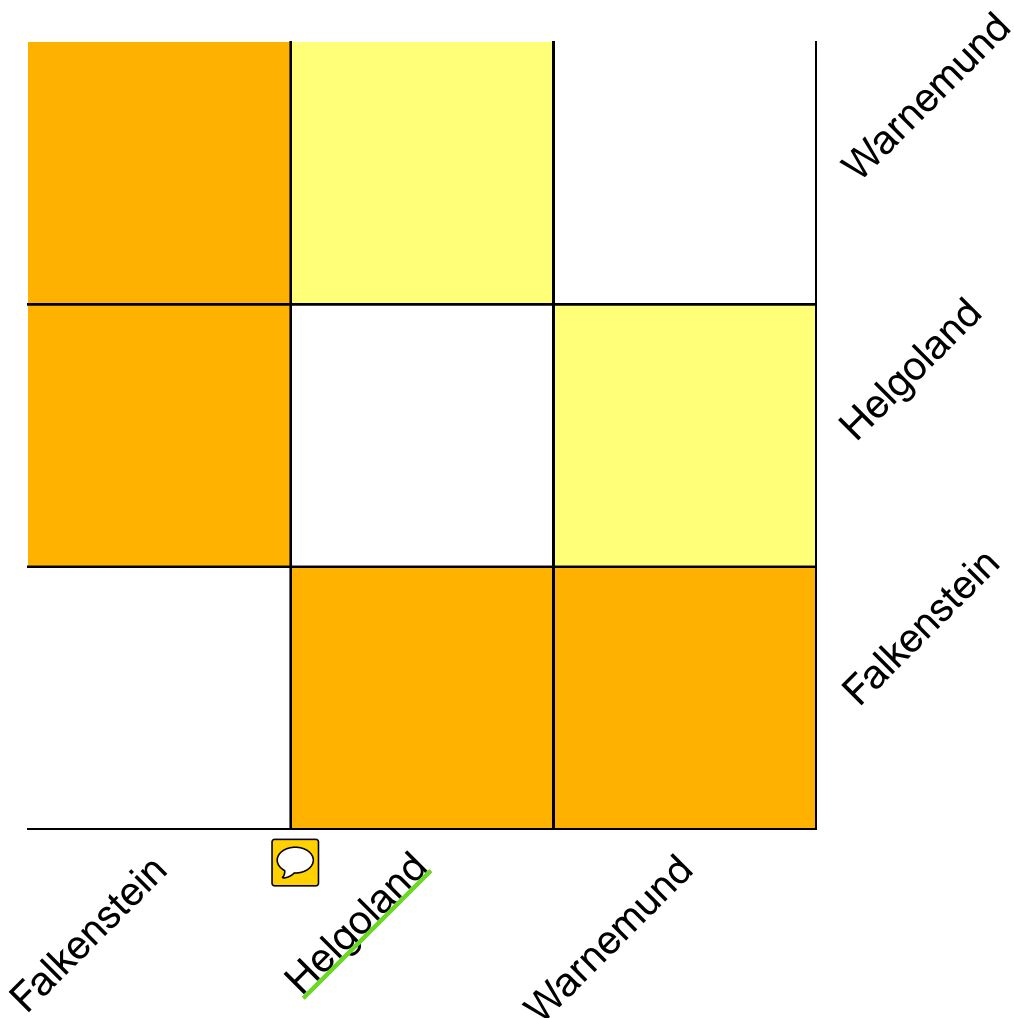

Supplement: Supplementary file 2 [file ECE3-7-7687-s002.pdf]

b)

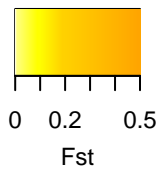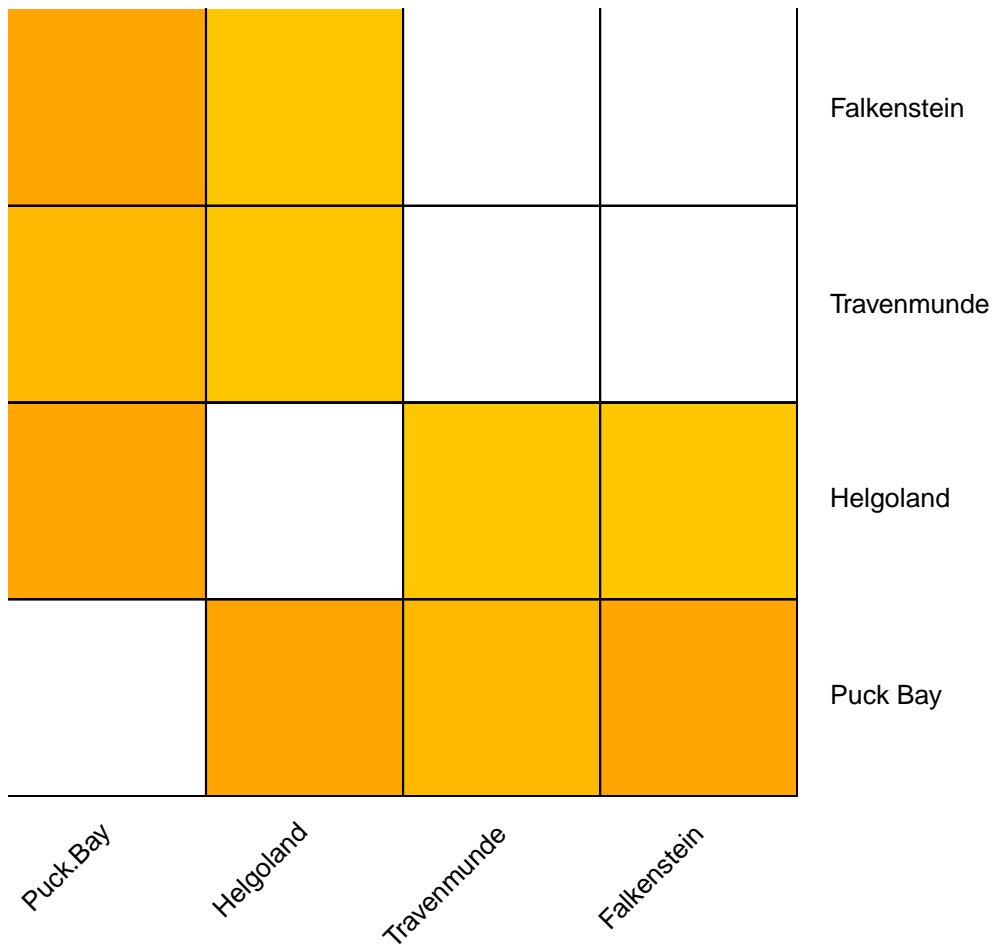

Supplement: Supplementary file 3 [file ECE3-7-7687-s003.pdf]

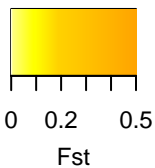

c)

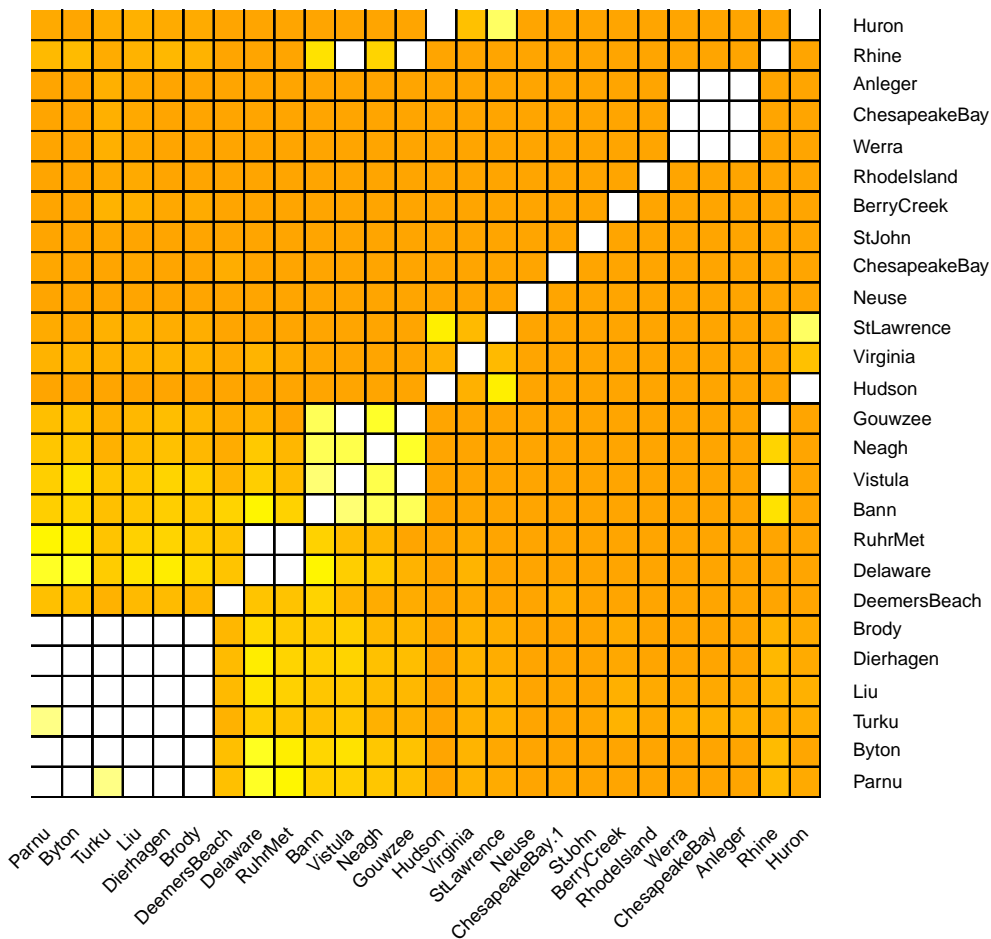

Supplement: Supplementary file 4 [file ECE3-7-7687-s004.pdf]

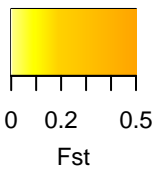

d)

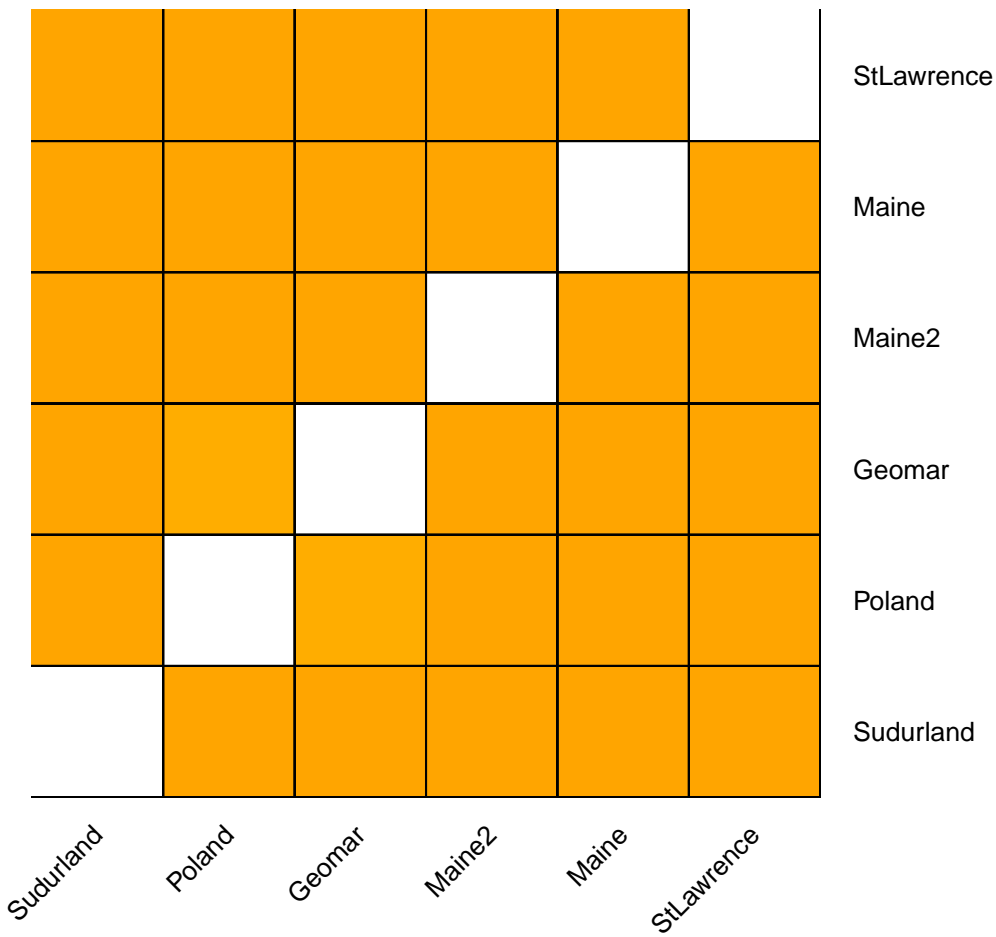

Supplement: Supplementary file 5 [file ECE3-7-7687-s005.pdf]

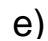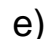

Supplement: Supplementary file 6 [file ECE3-7-7687-s006.pdf]

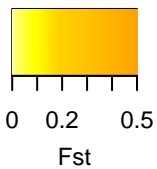

f)

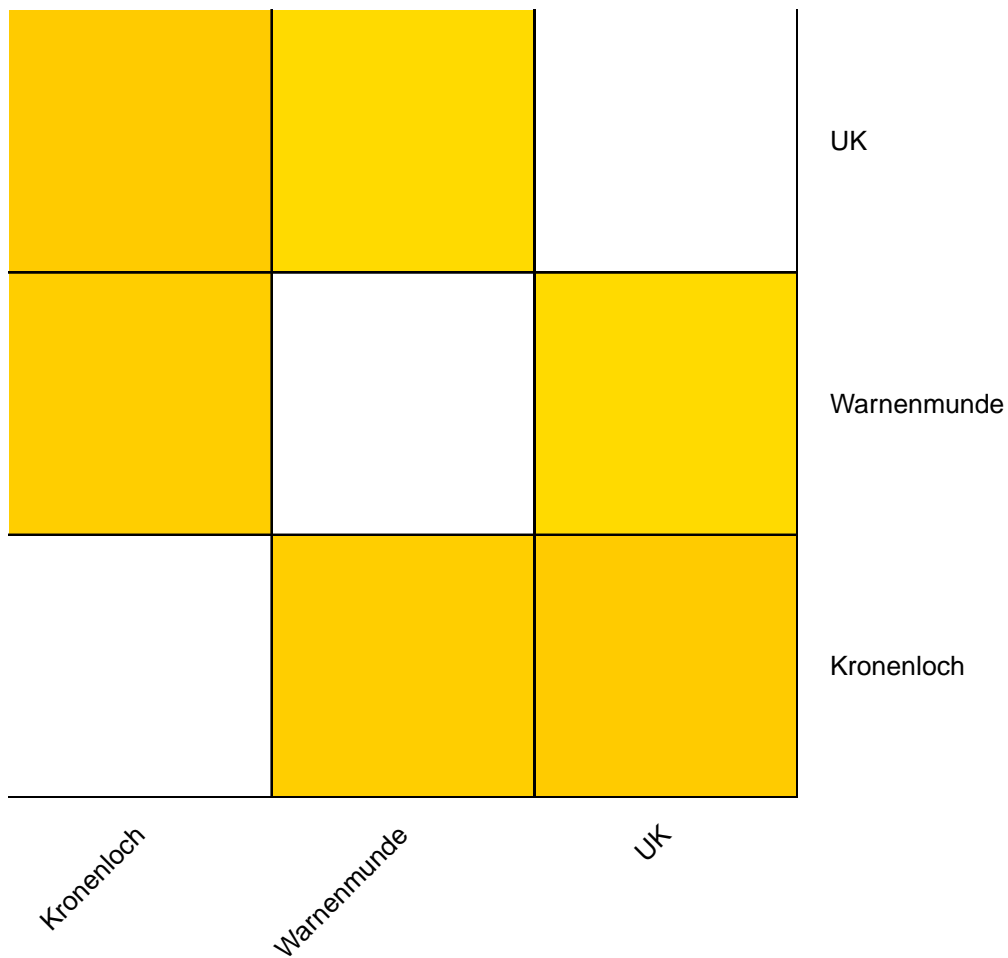

Supplement: Supplementary file 7 [file ECE3-7-7687-s007.pdf]
