# Supplementary material for: Diversity and distribution of genetic variation in gammarids: Comparing patterns between invasive and non‐invasive species
Source: Ecol Evol. 2017 Aug 22;7(19):7687–98. doi: 10.1002/ece3.3208 (PMC5632605; doi:10.1002/ece3.3208)
Supplement: Supplementary file 8 [file ECE3-7-7687-s008.docx]

Supplementary tables

| **species** | **individuals** | **locality** | **acession numbers** |
| --- | --- | --- | --- |
| *Gammarus locusta* | 28 | Falkenstein, Germany  54°39’ N; 10°19’ E | KU844859 - KU844872; KU844893 - KU844895; KU844898 - KU844908 |
|  | 24 | Helgoland,Germany  54°10'N; 7°53'E | KU844874 - KU844883;  KU844908 - KU844922 |
|  | 18 | Warnemünde,Germany, 54°13 N; 12°09’ E | KU844884 - KU844892 |
| *Gammarus oceanicus* | 14 | Kiel/Geomar,Germany, 54°32’ N; 10°14’ E | KU844924 - KU844928; KU844932; KU844935; KU844937; KU844943; KU844944; KU844946; KU844947- KU844951 |
|  | 12 | Maine, USA, 44°17‘ N; 67°33‘ W | FN675838 (5), FN675857, FN675858 (6) |
|  | 21 | Maine, USA, 43°36‘ N; 70°01‘ W | FN675840 (21) |
|  | 17 | St. Lawrence River, Canada, 49°02’ N; 67°57’ W | FN675859 (3), FN675860 (2), FN675861 (12) |
|  | 8 | Sudurland, Iceland, 64°04’ N; 22°71’ W | GQ341820, GQ341821, GQ341824, GQ341826- GQ341828, GQ341830, GQ341831 |
|  | 42 | Poland, 54°52‘ N; 17°02‘ E | FN675842, FN675843,FN675849, FN675850 (34), FN675851, FN675854, FN675855, FN675856 (2) |
| *Gammarus salinus* | 11 | Falkenstein, Germany, 54°40’ N; 10°20’ E | KU844968 - KU844978 |
|  | 15 | Helgoland,Germany, 54°10'N; 7°53'E |  |
|  | 14 | Travemünde, Germany, 53°83’ N; 10°64’ E | KU844979 - KU844981; KU844984 |
|  | 7 | Poland, Puck Bay, 54°72’ N; 18°41’ E | GQ341841- GQ341848 |
| *Gammarus zaddachi* | 24 | Warnemünde,Germany, 54°13 N; 12°09’ E | KU845051 - KU845056; KU845058 - KU845069 |
|  | 26 | Kronenloch, Germany, 54°09’ N; 8°96’ E | KU845070 - KU845095 |
|  | 5 | Wales, United Kingdom, 53°13 N; 4°09 W | GQ341862- GQ341866 |
| *Gammarus tigrinus* | 10 | Travemünde, Germany, 53°83’ N; 10°64’ E | KU844997 - KU845006 |
|  | 22 | Liu, Estonia, 58°27 N; 24°27 W | KU845009 - KU845027 |
|  | 19 | Pärnu, Estonia, 58°36 N; 24°46 W | KU845029 - KU845050 |
|  | 9 | St. John estuary, Canada, 45°37’ N; 66°15’ W | DQ300250 (8), DQ300251 |
|  | 24 | St. Lawrence, d/s Quebec, Cap Brule, Canada, 47°06’ N; 70°42’ W | DQ300211 (20), DQ300212 (4) |
|  | 7 | Lake Huron, Michigan, USA, 43°60’ N; 83°80’ W | DQ300211 (7) |
|  | 11 | Berrys creek, New Hampshire, USA, 43°04’ N; 70°73’ W | DQ300208 (3), DQ300209 (2), DQ300210 (6) |
|  | 6 | Delaware estuary, Delaware, USA, 39°57’ N; 75°58’ W | DQ300221, DQ300222 (4), DQ300223 |
|  | 19 | Delaware estuary, Deemers Beach, Delaware, USA, 39°64’ N; 75°59’ W | DQ523181 (12), DQ523182, DQ523183 (7), DQ523184, DQ523185 |
|  | 40 | Elizabeth estuary, Virginia, USA, 36°72’ N; 76°25’ W | DQ300224, DQ300225 (2), DQ300226- DQ300231, DQ523183 (7), DQ523186 (8), DQ523187, DQ523188 (2), DQ523189- DQ523191, DQ523192 (4), DQ523193, DQ523194 (4), DQ523195 |
|  | 25 | Hudson estuary, New York, USA, 40°96’ N; 73°90’ W | DQ300211 (24), DQ523179 (2) |
|  | 10 | Pawcatuck estuary, Rhode Island, USA, 41°33’ N; 71°83’ W | DQ300245 (5), DQ300246 (2), DQ300247- DQ300249 |
|  | 12 | Chesapeake Bay, Virginia,37°45’ N; 76°67’ W | DQ300219, DQ300220, DQ523180 (10), DQ523181 (3) |
|  | 9 | Neuse River: d/s New Bern, N. Carolina, USA, 35°08’ N; 77°57’ W | DQ300241- DQ300244, DQ300240 (6) |
|  | 10 | Turku, Finland, 60°40’ N ; 22°20’ E | DQ300212 (5), DQ523177, DQ523178, DQ523183 (3) |
|  | 10 | Vistula lagoon, Poland, 54°30’ N; 19°70’ E | DQ300212, DQ523183 (9) |
|  | 9 | Brody, Poland, 52°00’ N; 15°40’ E | DQ300212 (3), DQ523177 (4), DQ523178, DQ523183 (2) |
|  | 9 | Byton, Poland, 51°70’ N; 15°80’ E | DQ300212 (4), DQ523177 (2), DQ523178, DQ523183 (3) |
|  | 10 | Anleger Lagoon, Germany, 54°40’ N; 12°70’ E | DQ300212 (10) |
|  | 10 | Dierhagen lagoon, Germany, 54°20’ N ; 12°30’ E | DQ300212 (4), DQ523178 (3), DQ523183 (3) |
|  | 6 | Ruhr Metropolis, Germany | KT075215, KT075216, KT075217 (2), KT075218 (2) |
|  | 10 | Werra river, Germany, 51°30’ N; 9°70’ E | DQ300212 (10) |
|  | 10 | Lake Gouwzee, Netherlands, 52°40’ N; 5°00’ E | DQ300227, DQ523183 (9) |
|  | 9 | Bann river, North Ireland, 54°80’ N; 6°40’ W | DQ523178, DQ523181 (2), DQ523183 (7) |
|  | 12 | Lough Neagh, Northern Ireland, 54°70’ N; 6°50’ W | DQ523177, DQ523178 (3), DQ523183 (8) |
| *Obesogammarus crassus* | 14 | Gisom, Iran, 41°89’ N; 32°21’ E | KU845096 - KU845099; KU845108 - KU845110 |
|  | 18 | Havigh, Iran, 42°25’ N ; 31°71’ E | KU845100 - KU845107 |
|  | 9 | Chaboksar, Iran, 40°93’ N; 46°16’ E |  |
| *Pontogammarus maeoticus* | 22 | Shafarud, Iran, 38°21‘ N; 48°52‘ E | KU845119 - KU845122; KU845127;KU845128; KU845156- KU845160 |
|  | 29 | Bandare Anzali, Iran 37°65‘ N; 50°05‘ E | KU845111 - KU845114;  KU845123; KU845124; KU845144 - KU845155 |
|  | 22 | Jafrud, Iran 37°29‘ N; 49°30‘ E | KU845115 - KU845118; KU845125; KU845126; KU845129; KU845130; KU845132 - KU845137; KU845140; KU845141 |
|  | 7 | Sulina1, Ukraine, Black Sea, 45°17‘ N; 29°79‘ E | KC797063-KC797069 |
|  | 8 | Sulina2, Ukraine, Black Sea, 45°05‘ N; 29°67‘ E | KC797067, KC797070-KC797076 |
|  | 5 | Cape Kazantip, Azov Sea,45°45’ N; 36°00‘ E | KC797077-KC797079, KC797086-KC7970868 |
|  | 9 | Astara, Iran, Caspian Sea 39°03‘ N; 49°06‘ E | KC797075-KC797084 |
|  | 8 | Talesh, Iran, Caspian Sea 38°63‘ N; 49°02‘E | KC797075,KC797081,KC797085-KC7970819,KC797113 |
|  | 6 | Gisoom, Iran, Caspian Sea 38°18‘ N'; 49°05‘ E | KC797081,KC797083,KC797090-KC797093 |
|  | 7 | Bandare Anzali, Iran, Caspian Sea 37°65‘ N; 50°05‘ E | KC797075,KC797081,KC797082,KC797093-KC7970895,KF478575 |
|  | 6 | Kia Shahr, Iran, Caspian Sea, 37°51‘ N; 50°32‘ E | KC797081,KC797096-KC797098,KC797101,KC797102, |
|  | 6 | Motel Ghoo, Iran, Caspian Sea, 36°77‘ N; 51°17‘ E | KC797081,KC797097,KC797102,KC797103, |
|  | 6 | Noor, Iran, Caspian Sea 36°59‘ N; 52°02‘ E | KC797081,KC797097,KC797104,KC797105,KC797108,KC797109 |
|  | 8 | Mahmood Abad, Iran, Caspian Sea, 36°69‘ N; 51°80‘ E | KC797081,KC797097,KC797103-KC797108 |
|  | 8 | Khazar Abad, Iran, Caspian Sea,36°87‘ N; 53°14‘ E | KC797075,KC797081,KC797097,KC797099,KC797110-KC797113 |
| Total sequences used | 784 |  |  |
| Total populations screened | 59 |  |  |

**Table S1 – Information on the sequences used in this study.** List of the quantity, origin with GPS coordinates and NCBI accession number of the samples utilized in this study. “0” stands for sequences retrieved from NCBI, and “1” for sequences produced for this study.

| *G. locusta* | | | |
| --- | --- | --- | --- |
| Falckenstein | - | 0,003 | 0,001 |
| Helgoland | 0,01 | - | 0,003 |
| Waremünde | 0,006 | 0,011 | - |

**Table S2. Average estimates of pairwise evolutionary distances between sampling sites for *G locusta*.** The average number of base substitutions per site over all sequence pairs between sampling sites is shown in the lower diagonal; standard errors in the upper diagonal. Analyses were performed considering the species-specific substitution model.

| *G. salinus* | | | | |
| --- | --- | --- | --- | --- |
| Falckenstein | - | 0,003 | 0,002 | 0,003 |
| Helgoland | 0,013 | - | 0,003 | 0,005 |
| Travemünde | 0,01 | 0,016 | - | 0,003 |
| Puck Bay | 0,012 | 0,019 | 0,013 | - |

**Table S3. Average estimates of pairwise evolutionary distances between sampling sites for *G. salinus*.** The average number of base substitutions per site over all sequence pairs between sampling sites is shown in the lower diagonal; standard errors in the upper diagonal. Analyses were performed considering the species- specific substitution model.

| *G.oceanicus* | | | | | | |
| --- | --- | --- | --- | --- | --- | --- |
| Geomar | - | 0,006 | 0,007 | 0,002 | 0,007 | 0,003 |
| Maine1 | 0,022 | - | 0,005 | 0,006 | 0,003 | 0,006 |
| Maine2 | 0,024 | 0,015 | - | 0,007 | 0,005 | 0,007 |
| Poland | 0,003 | 0,024 | 0,026 | - | 0,007 | 0,003 |
| St,Lawrence | 0,025 | 0,007 | 0,014 | 0,027 | - | 0,007 |
| Sudurland | 0,004 | 0,021 | 0,024 | 0,006 | 0,025 | - |

**Table S4. Average estimates of pairwise evolutionary distances between sampling sites for *G. oceanicus*.** The average number of base substitutions per site over all sequence pairs between sampling sites is shown in the lower diagonal; standard errors in the upper diagonal. Analyses were performed considering the species-specific substitution model.

| *G. zaddachi* | | | |
| --- | --- | --- | --- |
| Waremünde | - | 0,002 | 0,003 |
| UK | 0,008 | - | 0,003 |
| Kronenloch | 0,015 | 0,015 | - |

**Table S5. Average estimates of pairwise evolutionary distances between sampling sites for *G. zaddachi*.** The average number of base substitutions per site over all sequence pairs between sampling sites is shown in the lower diagonal; standard errors in the upper diagonal. Analyses were performed considering the species- specific substitution model.

| *O. crassus* | | | |
| --- | --- | --- | --- |
| Gisom | - | 0,002 | 0,002 |
| Havigh | 0,008 | - | 0,002 |
| Chaboksar | 0,005 | 0,006 | - |

**Table S6. Average estimates of pairwise evolutionary distances between sampling sites for *O. crassus*.** The average number of base substitutions per site over all sequence pairs between sampling sites is shown in the lower diagonal; standard errors in the upper diagonal. Analyses were performed considering the species- specific substitution model.

| *G. tigrinus* | | | | | | | | | | | | | | | | | | | | | | | | | | |
| --- | --- | --- | --- | --- | --- | --- | --- | --- | --- | --- | --- | --- | --- | --- | --- | --- | --- | --- | --- | --- | --- | --- | --- | --- | --- | --- |
| Pärnu | - | 0,00 | 0,01 | 0,01 | 0,01 | 0,00 | 0,01 | 0,01 | 0,01 | 0,00 | 0,01 | 0,00 | 0,00 | 0,00 | 0,01 | 0,01 | 0,01 | 0,00 | 0,00 | 0,00 | 0,01 | 0,00 | 0,01 | 0,00 | 0,00 | 0,00 |
| Liu | 0,02 | - | 0,01 | 0,01 | 0,01 | 0,00 | 0,01 | 0,01 | 0,01 | 0,00 | 0,01 | 0,00 | 0,00 | 0,00 | 0,01 | 0,01 | 0,01 | 0,00 | 0,00 | 0,00 | 0,01 | 0,00 | 0,01 | 0,00 | 0,00 | 0,00 |
| Travemünde | 0,03 | 0,02 | - | 0,01 | 0,01 | 0,01 | 0,01 | 0,01 | 0,00 | 0,00 | 0,01 | 0,01 | 0,01 | 0,01 | 0,00 | 0,02 | 0,01 | 0,01 | 0,01 | 0,01 | 0,00 | 0,01 | 0,00 | 0,01 | 0,01 | 0,01 |
| StLawrence | 0,02 | 0,02 | 0,02 | - | 0,01 | 0,01 | 0,00 | 0,01 | 0,01 | 0,01 | 0,00 | 0,01 | 0,01 | 0,01 | 0,01 | 0,01 | 0,00 | 0,01 | 0,01 | 0,01 | 0,01 | 0,01 | 0,01 | 0,01 | 0,01 | 0,01 |
| ChesapeakBay | 0,02 | 0,02 | 0,04 | 0,02 | - | 0,00 | 0,01 | 0,01 | 0,01 | 0,01 | 0,01 | 0,01 | 0,00 | 0,00 | 0,01 | 0,01 | 0,01 | 0,01 | 0,01 | 0,01 | 0,01 | 0,01 | 0,01 | 0,01 | 0,01 | 0,01 |
| Delaware | 0,01 | 0,02 | 0,03 | 0,02 | 0,01 | - | 0,01 | 0,01 | 0,01 | 0,01 | 0,01 | 0,00 | 0,00 | 0,00 | 0,01 | 0,01 | 0,01 | 0,00 | 0,00 | 0,00 | 0,01 | 0,00 | 0,01 | 0,00 | 0,00 | 0,00 |
| Virginia | 0,02 | 0,02 | 0,03 | 0,01 | 0,02 | 0,02 | - | 0,01 | 0,01 | 0,01 | 0,00 | 0,01 | 0,00 | 0,01 | 0,01 | 0,01 | 0,00 | 0,01 | 0,01 | 0,01 | 0,01 | 0,01 | 0,01 | 0,01 | 0,01 | 0,01 |
| RhodeIsland | 0,03 | 0,02 | 0,01 | 0,02 | 0,03 | 0,03 | 0,03 | - | 0,01 | 0,01 | 0,01 | 0,01 | 0,01 | 0,01 | 0,00 | 0,01 | 0,01 | 0,01 | 0,01 | 0,01 | 0,01 | 0,01 | 0,01 | 0,01 | 0,01 | 0,01 |
| StJohn | 0,03 | 0,02 | 0,01 | 0,02 | 0,04 | 0,03 | 0,03 | 0,01 | - | 0,01 | 0,01 | 0,01 | 0,01 | 0,01 | 0,00 | 0,02 | 0,01 | 0,01 | 0,01 | 0,01 | 0,00 | 0,01 | 0,00 | 0,01 | 0,01 | 0,01 |
| Turku | 0,02 | 0,02 | 0,02 | 0,02 | 0,03 | 0,02 | 0,03 | 0,02 | 0,02 | - | 0,01 | 0,01 | 0,01 | 0,00 | 0,00 | 0,01 | 0,01 | 0,01 | 0,01 | 0,01 | 0,00 | 0,01 | 0,00 | 0,01 | 0,01 | 0,01 |
| Hudson | 0,02 | 0,02 | 0,02 | 0,00 | 0,02 | 0,02 | 0,01 | 0,02 | 0,02 | 0,02 | - | 0,01 | 0,01 | 0,01 | 0,01 | 0,01 | 0,00 | 0,01 | 0,01 | 0,01 | 0,01 | 0,01 | 0,01 | 0,01 | 0,01 | 0,01 |
| Bann | 0,02 | 0,02 | 0,04 | 0,02 | 0,02 | 0,01 | 0,02 | 0,03 | 0,04 | 0,02 | 0,02 | - | 0,00 | 0,00 | 0,01 | 0,01 | 0,01 | 0,00 | 0,00 | 0,00 | 0,01 | 0,00 | 0,01 | 0,00 | 0,00 | 0,00 |
| DeemersBeach | 0,02 | 0,02 | 0,04 | 0,02 | 0,01 | 0,01 | 0,02 | 0,03 | 0,04 | 0,02 | 0,02 | 0,01 | - | 0,00 | 0,01 | 0,01 | 0,01 | 0,00 | 0,00 | 0,00 | 0,01 | 0,00 | 0,01 | 0,00 | 0,00 | 0,00 |
| RurhMet | 0,01 | 0,02 | 0,04 | 0,02 | 0,01 | 0,01 | 0,02 | 0,03 | 0,03 | 0,02 | 0,02 | 0,01 | 0,01 | - | 0,01 | 0,01 | 0,01 | 0,00 | 0,00 | 0,00 | 0,01 | 0,00 | 0,01 | 0,00 | 0,00 | 0,00 |
| BerrysCreek | 0,02 | 0,02 | 0,01 | 0,02 | 0,03 | 0,03 | 0,03 | 0,01 | 0,01 | 0,02 | 0,02 | 0,03 | 0,03 | 0,03 | - | 0,01 | 0,01 | 0,01 | 0,01 | 0,01 | 0,00 | 0,01 | 0,00 | 0,01 | 0,01 | 0,01 |
| Neuse | 0,09 | 0,09 | 0,10 | 0,08 | 0,09 | 0,09 | 0,09 | 0,09 | 0,10 | 0,09 | 0,08 | 0,09 | 0,09 | 0,09 | 0,09 | - | 0,01 | 0,01 | 0,01 | 0,01 | 0,02 | 0,01 | 0,02 | 0,01 | 0,01 | 0,01 |
| LakeHuron | 0,02 | 0,02 | 0,02 | 0,00 | 0,02 | 0,02 | 0,01 | 0,02 | 0,02 | 0,02 | 0,00 | 0,02 | 0,02 | 0,02 | 0,02 | 0,08 | - | 0,01 | 0,01 | 0,01 | 0,01 | 0,01 | 0,01 | 0,01 | 0,01 | 0,01 |
| Vistula | 0,02 | 0,02 | 0,04 | 0,02 | 0,02 | 0,01 | 0,02 | 0,03 | 0,03 | 0,02 | 0,02 | 0,01 | 0,02 | 0,01 | 0,03 | 0,09 | 0,02 | - | 0,00 | 0,00 | 0,01 | 0,00 | 0,01 | 0,00 | 0,00 | 0,00 |
| Brody | 0,02 | 0,02 | 0,03 | 0,02 | 0,02 | 0,02 | 0,02 | 0,03 | 0,03 | 0,02 | 0,02 | 0,02 | 0,02 | 0,01 | 0,02 | 0,09 | 0,02 | 0,02 | - | 0,00 | 0,01 | 0,00 | 0,01 | 0,00 | 0,00 | 0,00 |
| Byton | 0,02 | 0,02 | 0,02 | 0,02 | 0,03 | 0,02 | 0,03 | 0,03 | 0,03 | 0,02 | 0,02 | 0,02 | 0,02 | 0,02 | 0,02 | 0,09 | 0,02 | 0,02 | 0,02 | - | 0,01 | 0,00 | 0,01 | 0,00 | 0,00 | 0,00 |
| Anleger | 0,03 | 0,02 | 0,00 | 0,02 | 0,04 | 0,03 | 0,03 | 0,01 | 0,01 | 0,02 | 0,02 | 0,04 | 0,04 | 0,04 | 0,01 | 0,10 | 0,02 | 0,04 | 0,03 | 0,02 | - | 0,01 | 0,00 | 0,01 | 0,01 | 0,01 |
| Dierhagen | 0,02 | 0,02 | 0,02 | 0,02 | 0,03 | 0,02 | 0,03 | 0,02 | 0,03 | 0,02 | 0,02 | 0,02 | 0,02 | 0,02 | 0,02 | 0,09 | 0,02 | 0,02 | 0,02 | 0,02 | 0,02 | - | 0,01 | 0,00 | 0,00 | 0,00 |
| Werra | 0,03 | 0,02 | 0,00 | 0,02 | 0,04 | 0,03 | 0,03 | 0,01 | 0,01 | 0,02 | 0,02 | 0,04 | 0,04 | 0,04 | 0,01 | 0,10 | 0,02 | 0,04 | 0,03 | 0,02 | 0,00 | 0,02 | - | 0,01 | 0,01 | 0,01 |
| Neagh | 0,02 | 0,02 | 0,04 | 0,02 | 0,02 | 0,01 | 0,02 | 0,03 | 0,04 | 0,02 | 0,02 | 0,01 | 0,01 | 0,01 | 0,03 | 0,09 | 0,02 | 0,01 | 0,01 | 0,02 | 0,04 | 0,02 | 0,04 | - | 0,00 | 0,00 |
| Rhine | 0,02 | 0,02 | 0,04 | 0,03 | 0,02 | 0,01 | 0,02 | 0,03 | 0,04 | 0,02 | 0,02 | 0,00 | 0,01 | 0,01 | 0,03 | 0,09 | 0,02 | 0,00 | 0,02 | 0,02 | 0,04 | 0,02 | 0,04 | 0,00 | - | 0,00 |
| Gouwzee | 0,02 | 0,02 | 0,04 | 0,02 | 0,02 | 0,01 | 0,02 | 0,03 | 0,04 | 0,02 | 0,02 | 0,00 | 0,01 | 0,01 | 0,03 | 0,08 | 0,02 | 0,01 | 0,02 | 0,02 | 0,04 | 0,02 | 0,04 | 0,00 | 0,00 | - |

**Table S7. Average estimates of pairwise evolutionary distances between sampling sites for *G. tigrinus*.** The average number of base substitutions per site over all sequence pairs between sampling sites is shown in the lower diagonal; standard errors in the upper diagonal. Analyses were performed considering the species- specific substitution model.

| *P. maeoticus* | | | | | | | | | | | | | | | |
| --- | --- | --- | --- | --- | --- | --- | --- | --- | --- | --- | --- | --- | --- | --- | --- |
| Bandare1 | - | 0,000 | 0,000 | 0,017 | 0,017 | 0,007 | 0,002 | 0,001 | 0,003 | 0,001 | 0,001 | 0,001 | 0,002 | 0,001 | 0,005 |
| Jafrud | 0,001 | - | 0,000 | 0,017 | 0,017 | 0,006 | 0,002 | 0,001 | 0,003 | 0,001 | 0,001 | 0,001 | 0,002 | 0,001 | 0,005 |
| Shafarud | 0,002 | 0,002 | - | 0,017 | 0,017 | 0,006 | 0,002 | 0,001 | 0,003 | 0,001 | 0,001 | 0,001 | 0,002 | 0,001 | 0,005 |
| Sulina1 | 0,113 | 0,114 | 0,113 | - | 0,002 | 0,017 | 0,015 | 0,017 | 0,014 | 0,017 | 0,017 | 0,017 | 0,015 | 0,017 | 0,013 |
| Sulina2 | 0,115 | 0,115 | 0,115 | 0,005 | - | 0,017 | 0,015 | 0,017 | 0,014 | 0,017 | 0,017 | 0,017 | 0,015 | 0,017 | 0,013 |
| Cape_Kazantip | 0,024 | 0,024 | 0,025 | 0,116 | 0,117 | - | 0,007 | 0,006 | 0,007 | 0,006 | 0,006 | 0,007 | 0,007 | 0,006 | 0,006 |
| Talesh | 0,017 | 0,018 | 0,018 | 0,100 | 0,101 | 0,036 | - | 0,003 | 0,004 | 0,003 | 0,003 | 0,002 | 0,004 | 0,003 | 0,006 |
| Gisoom | 0,002 | 0,003 | 0,003 | 0,113 | 0,115 | 0,024 | 0,018 | - | 0,003 | 0,001 | 0,001 | 0,001 | 0,003 | 0,001 | 0,005 |
| Bandare2 | 0,021 | 0,022 | 0,022 | 0,096 | 0,097 | 0,040 | 0,032 | 0,022 | - | 0,003 | 0,003 | 0,003 | 0,004 | 0,003 | 0,006 |
| Kia | 0,002 | 0,002 | 0,003 | 0,113 | 0,115 | 0,025 | 0,018 | 0,003 | 0,022 | - | 0,001 | 0,001 | 0,002 | 0,001 | 0,005 |
| Motel | 0,002 | 0,003 | 0,003 | 0,112 | 0,114 | 0,025 | 0,018 | 0,004 | 0,022 | 0,003 | - | 0,001 | 0,003 | 0,001 | 0,005 |
| Noor | 0,002 | 0,002 | 0,002 | 0,114 | 0,116 | 0,025 | 0,018 | 0,003 | 0,022 | 0,003 | 0,003 | - | 0,002 | 0,001 | 0,005 |
| Khazar | 0,016 | 0,017 | 0,017 | 0,099 | 0,100 | 0,037 | 0,028 | 0,017 | 0,031 | 0,017 | 0,017 | 0,017 | - | 0,003 | 0,006 |
| Mamhood | 0,002 | 0,003 | 0,003 | 0,114 | 0,115 | 0,026 | 0,018 | 0,004 | 0,022 | 0,003 | 0,004 | 0,003 | 0,018 | - | 0,005 |
| Astara | 0,037 | 0,038 | 0,038 | 0,087 | 0,088 | 0,043 | 0,044 | 0,038 | 0,046 | 0,038 | 0,038 | 0,038 | 0,044 | 0,038 | - |

**Table S8. Average estimates of pairwise evolutionary distances between sampling sites for *P. meioticus*.** The average number of base substitutions per site over all sequence pairs between sampling sites is shown in the lower diagonal; standard errors in the upper diagonal. Analyses were performed considering the species- specific substitution model.

| ***Species*** | ***Substitution model*** | ***lowest BIC*** | ***delta BIC*** |
| --- | --- | --- | --- |
| *G. locusta* | K2+G | 4988 | -3 |
| *G. salinus* | T92+G | 3462 | -8 |
| *G. tigrinus* | T92+G | 13090 | -2 |
| *G. oceanicus* | T92 | 5792 | -9 |
| *P. maeoticus* | T92+G | 6101 | -2 |
| *G. zaddachi* | T92+G | 3949 | -2 |
| *O. crassus* | T92 | 2803 | -4 |

**Table S9- Best-fit substitution models estimated for the different datasets.** Bayesian Inference Criteria (BIC) fits of 24 different nucleotide substitution modes. Total positions per dataset vary from species to species. Acronyms of each estimated substitution model mean the following: K2+G, Kimura 2-parameter with gamma distributions; T92+G, Tamura 3-parameters with gamma distribution; T92, Tamura 3-parameters.
